# Distributional Measures of Semantic Abstraction

**DOI:** 10.3389/frai.2021.796756

**Published:** 2022-02-08

**Authors:** Sabine Schulte im Walde, Diego Frassinelli

**Affiliations:** ^1^Institute for Natural Language Processing, University of Stuttgart, Stuttgart, Germany; ^2^Department of Linguistics, University of Konstanz, Konstanz, Germany

**Keywords:** lexical-semantic abstraction, abstractness, concreteness, generality, specificity, hypernymy, distributional semantics, vector spaces

## Abstract

This article provides an in-depth study of distributional measures for distinguishing between degrees of *semantic abstraction*. Abstraction is considered a “central construct in cognitive science” (Barsalou, 2003) and a “process of information reduction that allows for efficient storage and retrieval of central knowledge” (Burgoon et al., 2013). Relying on the distributional hypothesis, computational studies have successfully exploited measures of contextual co-occurrence and neighbourhood density to distinguish between conceptual semantic categorisations. So far, these studies have modeled semantic abstraction across lexical-semantic tasks such as ambiguity; diachronic meaning changes; abstractness vs. concreteness; and hypernymy. Yet, the distributional approaches target different conceptual types of semantic relatedness, and as to our knowledge not much attention has been paid to apply, compare or analyse the computational abstraction measures across conceptual tasks. The current article suggests a novel perspective that exploits variants of distributional measures to investigate semantic abstraction in English in terms of the abstract–concrete dichotomy (e.g., *glory–banana*) and in terms of the generality–specificity distinction (e.g., *animal–fish*), in order to compare the strengths and weaknesses of the measures regarding categorisations of abstraction, and to determine and investigate conceptual differences.

In a series of experiments we identify reliable distributional measures for both instantiations of lexical-semantic abstraction and reach a precision higher than 0.7, but the measures clearly differ for the abstract–concrete vs. abstract–specific distinctions and for nouns vs. verbs. Overall, we identify two groups of measures, (i) frequency and word entropy when distinguishing between more and less abstract words in terms of the generality–specificity distinction, and (ii) neighbourhood density variants (especially target–context diversity) when distinguishing between more and less abstract words in terms of the abstract–concrete dichotomy. We conclude that more general words are used more often and are less surprising than more specific words, and that abstract words establish themselves empirically in semantically more diverse contexts than concrete words. Finally, our experiments once more point out that distributional models of conceptual categorisations need to take word classes and ambiguity into account: results for nouns vs. verbs differ in many respects, and ambiguity hinders fine-tuning empirical observations.

## 1. Introduction

Over the years, interdisciplinary research on lexical semantics has seen multiple definitions of conceptual abstraction. For example, Barsalou (2003) considers abstraction as a “*central construct in cognitive science”* regarding categorical organisation in memory, and distinguishes between various types of abstraction. Burgoon et al. (2013) provide an extensive list and descriptions of past definitions of abstraction across research fields and research studies, and summarise the common core of abstraction types as “*a process of information reduction that allows for efficient storage and retrieval of central knowledge (e.g., categorization).”* Among the various types of abstraction described by Barsalou (2003) and Burgoon et al. (2013), we find two types that have repeatedly been connected to each other across disciplines, i.e., abstraction in terms of the abstract–concrete dichotomy (e.g., *glory* is more abstract than *banana*), and abstraction in terms of the generality–specificity distinction (e.g., *animal* is more abstract than *fish*). For example, one of the earliest datasets that collected abstractness ratings generated by humans was performed by Spreen and Schulz (1966), who in turn exploited two previously suggested tasks for abstractness ratings on a scale, to quantify abstractness (a) in contrast to concreteness in the sense of “*not perceived through senses,”* and (b) in contrast to specificity in the sense of “*general, generic.”* While the sense perception in task (a) was adopted as the standard task for collecting abstractness ratings in the following decades, these two categorisations demonstrate alternative instantiations of semantic abstraction, which were once more targeted in recent empirical studies. Theijssen et al. (2011) investigated annotations regarding (a) vs. (b) for noun senses in a corpus and for noun labels in dative alternations, and Bolognesi et al. (2020) correlated degrees of abstraction in collections of human-annotated concreteness vs. generality. Both studies were performed for English nouns and relied on existing norms of concreteness ratings (Coltheart, 1981; Brysbaert et al., 2014, respectively) and the hierarchical organisation of hypernymy in WordNet (Miller and Fellbaum, 1991; Fellbaum, 1998b).

In a similar manner but with yet different distinctions, we also find various instantiations of abstraction across sub-fields of computational lexical-semantic research. Relying on the distributional hypothesis that words which are similar in meaning also occur in similar linguistic distributions (Harris, 1954; Firth, 1957), these studies successfully exploited distributional measures of contextual co-occurrence and neighbourhood density to distinguish between conceptual semantic categorisations. For example, Sagi et al. (2009) applied a measure of neighbourhood density to quantify diachronic lexical semantic change; Hoffman et al. (2013) proposed semantic diversity as a measure of lexical semantic ambiguity; Santus et al. (2014) utilised the information-theoretic measure entropy to distinguish hypernyms from their hyponyms; Frassinelli et al. (2017) and Naumann et al. (2018) applied variants of neighbourhood density and entropy to distinguish between abstract and concrete words. While these studies address different lexical-semantic tasks, all tasks have in common that they involve and model some notion of semantic abstraction, i.e., diachronic innovative and reductive meaning change; lexical ambiguity; abstractness vs. concreteness in word meaning; and hypernymy. Yet, as to our knowledge, not much attention has been paid to the shared common meta-level task of quantifying abstraction across computational approaches, except for Rimell (2014) and Schlechtweg et al. (2017) using hypernymy measures for semantic entailment and diachronic change, respectively. Furthermore, a closer look into distributional neighbourhood variants reveals that the types of applied neighbourhoods are conceptually different, exploiting similarity between context words (Sagi et al., 2009; Hoffman et al., 2013; Naumann et al., 2018) vs. exploiting similarity between nearest neighbours (Frassinelli et al., 2017). In sum, most researchers involved in the respective sub-fields are not necessarily aware of each other, such that up to now we do not find a comprehensive application and comparison of distributional abstraction measures across semantic abstraction tasks.

The current article aims to fill this critical gap and provides a series of empirical studies that investigate conceptual categories of abstraction through variants of distributional measures. Focusing on the two types of abstraction originally suggested by Spreen and Schulz (1966), and brought back to attention by Theijssen et al. (2011) and Bolognesi et al. (2020), we distinguish abstraction in terms of the abstract–concrete dichotomy and in terms of the generality–specificity distinction. More specifically, we apply a selection of distributional measures to distinguish between English (i) abstract and concrete words and (ii) hypernyms and their hyponyms. As resources for our target words, we rely on the concreteness ratings in Brysbaert et al. (2014) and hypernymy relations in *WordNet* (Fellbaum, 1998b). Furthermore, we distinguish between noun and verb targets, given that lexical representations of word classes differ in their semantic abstraction regarding both concreteness and hypernymy (Miller and Fellbaum, 1991; Frassinelli and Schulte im Walde, 2019; Schulte im Walde, 2020). The specific measures we apply are variants of neighbourhood densities (context-based and neighbour-based), the distributional inclusion measure *WeedsPrec* (Weeds et al., 2014) and the information-theoretic measure *entropy* (Santus et al., 2014; Shwartz et al., 2017). The underlying distributional vector spaces are induced from the ENCOW web corpus (Schäfer and Bildhauer, 2012).

Overall, we thus suggest a novel perspective that brings together and effectively exploits empirical computational measures across two types of lexical-semantic abstraction. In this way, our studies enable us to compare the strengths and weaknesses of the distributional measures regarding categorisations of abstraction, and to determine and investigate conceptual differences as captured by the measures. In the remainder of this article, section 2 introduces previous research perspectives and studies on the two types of semantic abstraction we focus on, both from a cognitive and from a computational perspective. Section 3 then describes the data and methods we use in our study, before section 4 provides the actual experiments and results which are then discussed in section 5.

## 2. Related Work

In the following, we introduce previous research perspectives and studies on the two types of semantic abstraction we focus on, i.e., abstraction in terms of the abstract–concrete dichotomy and in terms of the generality–specificity distinction. In this vein, section 2.1 looks into abstraction from a cognitive perspective, while section 2.2 provides an overview of computational models of abstraction. In section 2.3, we describe previous empirical investigations across the two types of abstraction. From a terminological perspective, we will use the word “concepts” when referring to mental representations, and “words” when referring to the corresponding linguistic surface forms humans are exposed to. Given the distributional nature of our studies, we will always refer to words as the targets of our analyses.

### 2.1. Cognitive Perspectives on Abstraction

Barsalou (2003) considers abstraction as a “*central construct in cognitive science”* regarding the organization of categories in the human memory. He attributes six different senses to abstraction: (i) abstracting a conceptual category from the settings it occurs in; (ii) generalising across category members; (iii) generalising through summary representations which are necessary for the behavioural generalisations in (ii); (iv) sparse schematic representations; (v) flexible interpretation; and (vi) abstractness in contrast to concreteness. Barsalou's classification illustrates that the term “semantic abstraction” as well as its featural and inferential implications for memory representations are vague in that different instantiations go along with different representations; he himself focuses on summary representations (iii). Burgoon et al. (2013) provide an extensive list and description of past definitions of abstraction across research fields and research studies, and state that, at the meta level, the term abstraction is referred to as “*a process of information reduction that allows for efficient storage and retrieval of central knowledge (e.g., categorization).”* For their own study, they define abstraction as “*as a process of identifying a set of invariant central characteristics of a thing,”* and in what follows they compare existing definitions of abstraction regarding their roots, developments, antecedents, consequences, and methods for studying.

The distinction of the two abstraction types adopted in the current study comes from Spreen and Schulz (1966) indicating that the “*definition of abstractness or concreteness in previous studies shows that at least two distinctly different interpretations can be made,”* and pointing back to previous collections with judgements on generality by Gorman (1961) and judgements on concreteness as well as generality by Darley et al. (1959). Spreen and Schulz (1966) themselves collected ratings on both abstractness–concreteness and abstractness–specificity (among others) for 329 English nouns, and found a correlation of 0.626 between the ratings of the two abstraction variables. The two-fold distinction of abstraction outlined in the work by Spreen and Schulz (1966) is also included in the various instantiations of abstraction in Barsalou (2003) and Burgoon et al. (2013). In the following, we describe the lines of research involved in the representation and processing of abstract vs. concrete concepts and then those involved in general vs. specific concepts.

#### 2.1.1. Abstract vs. Concrete Concepts

The most influential proposal about the processing, storing and comprehension of abstract concepts in contrast to concrete concepts can be traced back to Paivio (1971). He suggested the *dual-route theory* where a verbal system is primarily responsible for language aspects of linguistic units (such as words), while a non-verbal system, in particular imagery, is primarily responsible for sensory-motor aspects. Even though in the meantime, a range of alternative as well as complementary theories have been suggested, Paivio's theory offers an explanation why concrete concepts (which are supposedly accessed via both routes) are generally processed faster in lexical memory than abstract concepts (which are supposedly accessed only via the non-verbal system) across tasks and datasets, cf. Pecher et al. (2011) and Borghi et al. (2017) for comprehensive overviews.

Further than the dual-route theory, cognitive scientists have investigated other dimensions of abstractness. Most notably, Schwanenflugel and Shoben (1983) suggested the *context availability theory* where they compared the processing of abstract and concrete words in context and demonstrated that in appropriate contexts neither reading times nor lexical decision times differ, thus emphasising the role of context in conditions of abstractness. In addition, a number of properties have been pointed out where abstract and concrete concepts differ. (i) There is a strong consensus and experimental confirmation that concrete concepts are more *imaginable* than the abstract ones, and that it takes longer to generate images for abstract than for concrete concepts (Paivio et al., 1968; Paivio, 1971; Paivio and Begg, 1971, i.a.). (ii) Abstract concepts are considered to be more *emotionally valenced* than concrete concepts (Kousta et al., 2011; Vigliocco et al., 2014; Pollock, 2018). (iii) *Free associations* to abstract concepts are assumed to differ from free associations to concrete concepts in terms of the number of types, but at the same time associations to concrete concepts have been found weaker and more symmetric than for abstract concepts (Crutch and Warrington, 2010; Hill et al., 2014). (iv) Based on a *feature generation task*, features of abstract concepts are less property- and more situation-related than features of concrete words (Wiemer-Hastings and Xu, 2005). (v) Accordingly, an appropriate embedding into *situations* has been identified as crucial for abstract vs. concrete meaning representations (Barsalou and Wiemer-Hastings, 2005; Hare et al., 2009; Pecher et al., 2011; Frassinelli and Lenci, 2012; Recchia and Jones, 2012).

Hand in hand with defining and investigating hypotheses about dimensions of abstract and concrete concepts, a number of data collections have been created. To name just a prominent subset of the large number of existing resources, Spreen and Schulz (1966) collected ratings of concreteness and specificity (among others) for 329 English nouns (see above); Paivio et al. (1968) collected ratings for 925 English nouns on concreteness, imagery and meaningfulness; Coltheart (1981) put together the *MRC Psycholinguistic Database*, mostly comprising pre-existing information for almost 100,000 English words including concreteness, imageability, familiarity as well as frequency, semantic, syntactic, and phonological information; Warriner et al. (2013) extended the *ANEW* norms from Bradley and Lang (1999) with 1,034 English words to almost 14,000, capturing emotion-relevant norms of valence, arousal and dominance; a similar collection for 20,000 English words regarding the same variables but using best–worst scaling instead of ratings has been done by Mohammad (2018); Brysbaert et al. (2014) created the so far largest human-generated collection containing concreteness ratings for 40,000 English words. The work by Connell and Lynott differs slightly on the variable depth, by focusing on the individual perception modalities and interoception (Lynott and Connell, 2009, 2013; Lynott et al., 2020). While the vast amount of abstractness/concreteness datasets has been created for English, we also find collections for other languages, such as those for 2,654/1,000 nouns in German (Lahl et al., 2009; Kanske and Kotz, 2010, respectively); 16,109 Spanish words (Algarabel et al., 1988); 417 Italian words (Della Rosa et al., 2010); and 1,659 French words (Bonin et al., 2018). While traditional collections have been pen-and-paper-based, the collections from the last decade have moved toward crowd-sourcing platforms. As alternative to human-generated ratings, previous research suggested semi-automatic algorithms to create large-scale norms (Mandera et al., 2015; Recchia and Louwerse, 2015; Köper and Schulte im Walde, 2016; Köper and Schulte im Walde, 2017; Aedmaa et al., 2018; Rabinovich et al., 2018).

#### 2.1.2. General vs. Specific Concepts

Differently to the above distinction of semantic abstraction in terms of degrees of concreteness as opposed to abstractness, where concepts may be judged more or less abstract in comparison to otherwise semantically unrelated concepts (e.g., *banana–glory*), semantic abstraction in terms of generality is typically established in contrast to a semantically related concept (e.g., *animal–fish*). The lexical-semantic relation of interest here is hypernymy, where the more general concept represents the hypernym of the more specific hyponym.

An enormous body of work discusses hypernymy next to further semantic relations in the mental lexicon. For example, a seminal description of lexical relations can be found in Cruse (1986), who states that lexical relations “*reflect the way infinitely and continuously varied experienced reality is apprehended and controlled through being categorised, subcategorised and graded along specific dimensions of variation.”* Murphy (2003) focuses on the representation of semantic relations in the lexicon and discusses synonymy, antonymy, contrast, hyponymy and meronymy, across word classes. Most of her discussions concern linguistic vs. meta-linguistic representations of relations, reference of relations to words vs. concepts, and lexicon storage. The most extensive resource that systematically explores and compares types of lexical-semantic relations across word classes is established by the taxonomy of the Princeton *WordNet*, where hypernymy represents a key organisation principle of semantic memory (Fellbaum, 1990; Gross and Miller, 1990; Miller et al., 1990). Miller and Fellbaum (1991) provide a meta-level summary of relational structures and decisions. As basis for the WordNet organisation, they state that “*the mental lexicon is organised by semantic relations. Since a semantic relation is a relation between meanings, and since meanings can be represented by synsets, it is natural to think of semantic relations as pointers between synsets.”* The semantic relations in WordNet include the paradigmatic relations synonymy, hypernymy/hyponymy, antonymy, and meronymy. For nouns, WordNet implements a hierarchical organisation of synsets (i.e., sets of synonymous word meanings) relying on hypernymy relations. Verbs are considered the most complex and polysemous word class; they are organised on a verb-specific variant of hypernymy, i.e., *troponymy:* *v*_1_ *is to* *v*_2_ *in some manner*, that operates on semantic fields instantiated through synsets. Troponymy itself is conditioned on entailment and temporal inclusion.

### 2.2. Computational Models of Abstraction

Across both types of semantic abstraction, computational models have been suggested to automatically characterise or distinguish between more and less abstract words. They have been intertwined with cognitive perspectives to various degrees.

#### 2.2.1. Abstract vs. Concrete Words

A common idea in this research direction is the exploitation of corpus-based co-occurrence information to infer textual distributional characteristics of cognitive semantic variables, including abstractness as well as further variables such as emotion, imageability, familiarity, etc. These models are large-scale data approaches to explore the role of linguistic information and textual attributes when distinguishing between abstract and concrete words. A subset of these distributional approaches is strongly driven by a cognitive perspective, thus aiming to explain the organisation of human semantic memory and lexical processing effects by the contribution of linguistic attributes. Common techniques for organising the textual information are semantic vector spaces such as Latent Semantic Analysis (LSA) (Salton et al., 1975), the Hyperspace Analogue to Language (HAL) (Burgess, 1998), and more recent variants of standard Distributional Semantic Models (DSMs) (Baroni and Lenci, 2010; Turney and Pantel, 2010), in combination with measures of distributional similarity and clustering approaches (Glenberg and Robertson, 2000; Vigliocco et al., 2009; Bestgen and Vincze, 2012; Troche et al., 2014; Mandera et al., 2015; Recchia and Louwerse, 2015; Lenci et al., 2018). Finally, our own studies provide preliminary insights into co-occurrence characteristics of abstract and concrete words with respect to linguistic parameters such as window size, parts-of-speech and subcategorisation conditions (Frassinelli et al., 2017; Naumann et al., 2018; Frassinelli and Schulte im Walde, 2019). Overall, these studies agree on tendencies such that concrete words tend to have less diverse but more compact and more strongly associated distributional neighbours than abstract words.

#### 2.2.2. General vs. Specific Words

From a computational perspective, hypernymy—which we take as instantiation to represent degrees of generality vs. specificity—is central to solving a number of NLP tasks such as automatic taxonomy creation (Hearst, 1998; Cimiano et al., 2004; Snow et al., 2006; Navigli and Ponzetto, 2012) and textual entailment (Dagan et al., 2006; Clark et al., 2007). An enormous body of computational work has applied variants of lexico-syntactic patterns in order to distinguish hypernymy among word pairs from other lexical semantic relations (Hearst, 1992; Pantel and Pennacchiotti, 2006; Yap and Baldwin, 2009; Schulte im Walde and Köper, 2013; Roth and Schulte im Walde, 2014; Nguyen et al., 2017, i.a.). More closely related to the current study, Shwartz et al. (2017) provide an extensive overview and comparison of unsupervised distributional methods. They distinguish between families of distributional approaches, i.e., *distributional similarity measures* (assuming asymmetric distributional similarities for hypernyms and their hyponyms regarding their contexts, e.g., Santus et al., 2016), *distributional inclusion measures* (comparing asymmetric directional overlap of context words, e.g., Weeds and Weir, 2005; Kotlerman et al., 2010; Lenci and Benotto, 2012) and *distributional informativeness measures* (assuming different degrees of contextual informativeness, e.g., Rimell, 2014; Santus et al., 2014). Across modelling systems, most approaches model hypernymy between nouns; hypernymy between verbs has been addressed less extensively from an empirical perspective (Fellbaum, 1990, 1998a; Fellbaum and Chaffin, 1990).

### 2.3. Empirical Models Across Types of Abstraction

In addition to interdisciplinary empirical research targeting concreteness or hypernymy that has been mentioned above, we find at least two empirical studies at the interface of cognitive and computational linguistics that brought together our two target types of abstraction beforehand, Theijssen et al. (2011) and Bolognesi et al. (2020). Similarly to the current work, Theijssen et al. (2011) used the observation in Spreen and Schulz (1966) defining abstraction in terms of concreteness and specificity as their starting point. They provide two empirical experimental setups to explore and distinguish between the abstraction types in actual system implementations, (1) based on existing annotations of noun senses in a corpus, and (2) based on human judgements on labelling nouns in English dative alternations. As resources they used the MRC database (Coltheart, 1981) and WordNet. Overall, they found cases where concreteness and specificity overlap and cases were the two types of abstraction diverge. Bolognesi et al. (2020) looked into the same two types of abstraction to correlate degrees of abstraction in the concreteness norms by Brysbaert et al. (2014) and in the WordNet hierarchy, and to investigate interactions between the four groups of more/less concrete × more/less specific English nouns from the two resources. Their studies illustrate that concreteness and specificity represent two distinct types of abstraction.

Further computational approaches zoomed into statistical estimation of contextual diversity/neighbourhood density, in order to distinguish between degrees of semantic abstraction across types of abstraction. For example, McDonald and Shillcock (2001) applied the information-theoretic measure *relative entropy* to determine the degree of informativeness of words, where word-specific probability distributions over contexts were compared with distributions across corresponding sets of words. The contextual diversity measure by Adelman et al. (2006) is comparably more simple: they determined the number of documents in a corpus that contain a word. More recently, Danguecan and Buchanan (2016), Reilly and Desai (2017) and our own work in Naumann et al. (2018) explored variants of neighbourhood density measures for abstract and concrete words, i.e., the number of (different) context words and the distributional similarity between context words. Additional approaches to determine contextual diversity/neighbourhood density have arisen from other fields of research concerned with semantic abstraction, i.e., regarding ambiguity and diachronic meaning change (Sagi et al., 2009; Hoffman et al., 2013; Hoffman and Woollams, 2015). Overall, these studies demonstrated that contextual density/diversity differs for more vs. less abstract words and across types of abstraction, even though the applications of the measures were rather diverse.

## 3. Materials and Methods

### 3.1. Abstraction Data: Concreteness and Hypernymy

In the following, we introduce the resources we used for creating variants of abstraction data for our distributional experiments in section 4. As motivated above, we distinguish semantic abstraction in terms of the abstract–concrete and the generality–specificity distinctions.

#### 3.1.1. Concreteness Targets

Regarding abstraction in terms of the abstract–concrete dichotomy (henceforth referred to as **concreteness** condition), we rely on the concreteness ratings for approximately 40,000 English words and two-word expressions from Brysbaert et al. (2014). The ratings were collected via Amazon Mechanical Turk by asking at least 25 participants to judge the concreteness vs. abstractness of the targets on a 5-point rating scale from 1 (abstract) to 5 (concrete) regarding how strongly the participants thought the meanings of the targets can(not) be experienced directly through their five senses. The overall targets' scores of abstractness vs. concreteness are represented by the mean values. For example, the concrete word *banana* received the highest possible average rating of 5.0 because it is strongly perceived by human senses, while the abstract word *glory* received a rather low average rating of 1.45.

The ratings had been collected for the targets out-of-context and without any further word-class disambiguating information. In a post-processing step, Brysbaert et al. added part-of-speech (POS) and frequency information from the SUBTLEX-US corpus (Brysbaert et al., 2012). We repeated their post-processing step, however relying on the ENCOW corpus data we also use in our studies (see below for details), i.e., we automatically assigned each target its most frequently occurring POS tag in the ENCOW.

**Figure 1:**
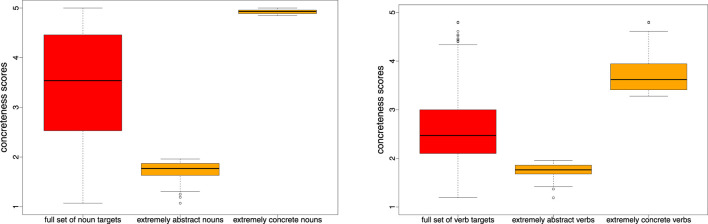
Distributions of concreteness scores on a 5-point rating scale from 1 (abstract) to 5 (concrete) for our full concreteness sets of 5,448/1,280 nouns/verbs and for the 500/200 most extreme abstract and concrete nouns/verbs.

If this POS did not represent an overall proportion of at least 95% of all POS tags of that target or if our most-frequent POS was not identical to the POS tag assigned by Brysbaert et al. (2014), we discarded the target in order to minimise POS ambiguity among targets. We also discarded target words with an ENCOW frequency below 10,000. Our final concreteness set of targets contains 5,448 nouns and 1,280 verbs. Henceforth, we will refer to this selection of datapoints as the **full concreteness** collection. We also created target subsets of the 500 most concrete and the 500 most abstract nouns, and ditto for the 200 most concrete/abstract verbs. We will refer to these subsets as the **concreteness extremes** subsets. Figure 1 illustrates the distributions of concreteness scores across the full and extreme target sets; the underlying files are provided in the Supplementary Material.

#### 3.1.2. Hypernymy Targets

Regarding abstraction in terms of generality (henceforth referred to as **hypernymy** condition), we rely on WordNet, a standard lexical semantic taxonomy for English developed at Princeton University (Miller and Fellbaum, 1991; Fellbaum, 1998b) that was also used by previous work on the generality–specificity abstraction distinction (Theijssen et al., 2011; Bolognesi et al., 2020). The lexical database was inspired by psycholinguistic research on human lexical memory and organises English nouns, verbs, adjectives and adverbs into classes of synonyms *(synsets)*, which are connected by lexical and conceptual semantic relations. Words with several senses are assigned to multiple synsets. As mentioned above, WordNet implements a hierarchical organisation of noun synsets relying on hypernymy relations, and verbs are organised by a verb-specific variant of hypernymy, i.e., *troponymy:* *v*_1_ *is to* *v*_2_ *in some manner*, which itself is conditioned on entailment and temporal inclusion.

**Figure 2:**
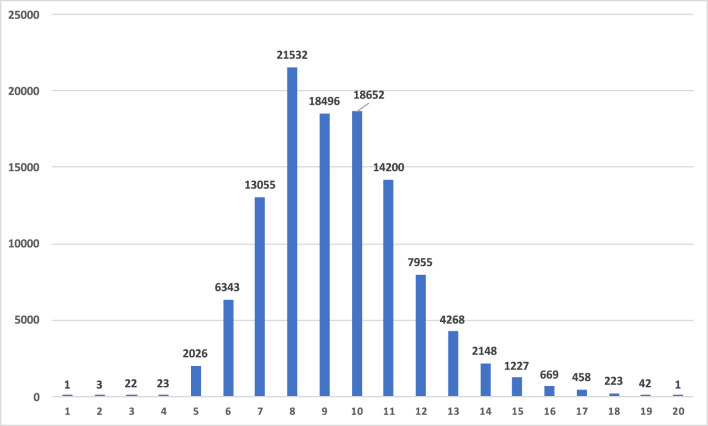
Number of synsets per hypernymy level in the WordNet noun hierarchy, with level 1 representing the top-most and therefore most general synset {*entity*}.

We extracted all noun and verb synset pairs from WordNet version 3.0 that are in a hyponym–hypernymy relation and paired all nouns/verbs from the respective subsets (such as *trout–fish* and *swim–move*, where the first word in the pairs is the semantically more specific hyponym and the second word in the pairs is the semantically more general hypernym), resulting in a total of 295,963/67,586 word pairs for nouns/verbs. We then discarded any pairs containing multiword targets (such as *edible fruit*) as well as targets starting with a capital letter (mostly proper names such as *Xhosa*) or starting with a number, leaving a total of ≈110,000/47,500 noun/verb pairs containing ≈38,000/8,500 different nouns/verbs. Figure 2 shows the number of synsets per level in the noun hierarchy, with level 1 representing the top-most and therefore most general synset {*entity*}. For verbs this analysis is not straightforward, as many synsets do not have a hypernym, and the top levels are not consistently connected downwards [also see Richens (2008) on “anomalies in the WordNet verb hierarchy”]; this is the reason why some hypernymy-level-related analyses in section 4 will not be performed for verbs.

### 3.2. Vector Space Variants

The basis for our experiments is represented by the POS-tagged version of the sentence-shuffled English COW corpus ENCOW16AX^1^, containing ≈10 billion words (Schäfer and Bildhauer, 2012; Schäfer, 2015). From the corpus, we extracted co-occurrences (i.e., context words) for all nouns and verbs in the corpus by applying a standard range of co-occurrence options: We relied on 2-word and 20-word symmetric windows (left+right) across the lemmatised version of the corpus and distinguished between (a) taking only co-occurring noun context words into account (henceforth: N space) and (b) taking all co-occurring nouns, verbs and adjectives into account (henceforth: N-V-A space), when creating our noun–context and verb–context matrices. The windows were applied within-sentence because the corpus is sentence-shuffled for copyright reasons, such that going beyond sentence border is not meaningful. Furthermore, to reduce noise in the co-occurrence data, we restricted the corpus lemmas to words starting with at least two letters; by using a co-occurrence frequency cut-off of 50; and by discarding the most frequent content words: *people, time, year* (nouns); *be, do, have* (verbs); and *other, more, many, such, same, few, most* (adjectives), given that high-frequency words are notorious hubs and popular nearest neighbours in the vector spaces (Radovanović et al., 2010; Dinu et al., 2015; Köper et al., 2016, i.a.). The raw co-occurrence frequency counts were weighted by the association measure *local mutual information (lmi)*, cf. Evert (2005). LMI is an information-theoretic association measure that compares observed frequencies *O* with expected frequencies *E*, taking marginal frequencies into account: $LMI=O\times log\frac{O}{E}$, with *E* representing the product of the marginal frequencies over the sample size.^2^

Our co-occurrence matrices are general-purpose and not prone to our specific resource-induced targets, which is required by some abstraction measures (see following section 3.3). Table 1 shows the sizes of our vector space matrix variants in numbers of targets and dimensions, i.e., context words. Table 2 shows co-occurrence frequencies and lmi scores for a sample noun, i.e., *fish*, and a selection of its context words within a window of ±20 words.

**Table 1:** Sizes of vector space variants in terms of numbers of target types and dimension types in the co-occurrence (context) matrices.

**Target POS**	**Window size**	**Dimension POS**	**# targets**	**# dimensions**
N	2	N	22,017	22,017
		N-V-A	24,279	40,571
	20	N	29,721	29,721
		N-V-A	30,748	51,249
V	2	N	6,259	16,373
		N-V-A	6,544	28,736
	20	N	7,338	25,254
		N-V-A	7,530	43,329

**Table 2:** Example context words for the target noun *fish* within a window of ±20 words, accompanied by co-occurrence frequencies and local mutual information (lmi) scores.

**Context word and POS**		**Frequency**	**LMI**
water	NN	56,049	133387.53
tank	NN	39,118	150223.00
catch	V	37,003	117624.73
eat	V	31,558	87119.87
small	ADJ	30,864	45470.63
big	ADJ	24,835	37067.61
chip	NN	19,407	72473.17
oil	NN	18,404	41075.69
salmon	NN	8,983	38461.76
tropical	ADJ	6,629	23600.64
serve	V	6,571	4433.21
eye	NN	4,052	1701.02

### 3.3. Abstraction Measures

The following subsections introduce our selection of distributional methods to measure abstraction both in terms of the abstractness–concreteness dichotomy and in terms of the generality–specificity distinction.

#### 3.3.1. Neighbourhood Densities

Our main focus regarding vector space measures of abstraction lies on variants of neighbourhood densities. As described in section 2, previous work has applied such measures to a number of tasks involving semantic abstraction (not necessarily using the identical term “neighbourhood density”), such as lexical semantic ambiguity (Hoffman et al., 2013), lexical semantic change (Sagi et al., 2009), hypernymy (Santus et al., 2014) and lexical concreteness (Frassinelli et al., 2017; Naumann et al., 2018). The underlying assumption of the empirical models across tasks is that the neighbourhood density of more abstract words is lower than the neighbourhood density of less abstract (i.e., more specific/concrete) words, because conceptual connections between abstract words and their semantically associated words are more diverse/variable and less meaning-specific than conceptual connections between more specific/concrete words and their semantically associated words.

In this vein, neighbourhood density measures score the variability of contexts in which words occur in different ways. They either (i) measure neighbourhood density by relying on **context words**, assuming that more abstract words co-occur with a larger variety of **context words**, or they (ii) measure neighbourhood density by relying on **neighbour words**, assuming that more abstract words have a larger variety of distributionally similar words. As mentioned above, these types of neighbourhood densities are conceptually rather different, exploiting similarity between **context words** vs. exploiting similarity between nearest neighbours. In addition, neighbourhood density measures differ with respect to involving (or not involving) the respective target words in the calculation. Finally, all variants of measures need to define the number *k* of context/neighbour words that are taken into account, i.e., how many words are involved as “strongest” context/neighbour words. The four variants are defined and computed as follows.

CC The neighbourhood density of a target word *t* is defined as the average vector-space distance **between the** ***k*** **strongest context words** of *t*.
TC The neighbourhood density of a target word *t* is defined as the average vector-space distance **between** ***t*** **and its** ***k*** **strongest context words**.
NN The neighbourhood density of a target word *t* is defined as the average vector-space distance **between the** ***k*** **nearest neighbours of** ***t***.
TN The neighbourhood density of a target word *t* is defined as the average vector-space distance **between** ***t*** **and its** ***k*** **nearest neighbours**.

The strongest context words are determined on the basis of the local mutual information strength of co-occurrence (see previous section 3.2). Vector-space distance between words in order to determine nearest neighbours is computed by calculating the *cosine* of the angle between the respective word vectors. See Supplementary Table 1 in Appendix 1 for examples of strongest context and neighbour words regarding a selection of target nouns and verbs.

#### 3.3.2. Contextual Entropy

For measuring the contextual entropy of a target word we rely on standard word entropy, which has been suggested as an asymmetric method for hypernymy prediction by Shwartz et al. (2017), inspired by a previous second-order co-occurrence variant (Santus et al., 2014). The underlying assumption is that more abstract words are more uncertain (and therefore receive a higher entropy value) than less abstract (i.e., more specific/concrete) words. For each target word *w* in our vector spaces we calculated the word entropy *H*(*w*), taking all of *w*'s context words *c* from our vector spaces into account, see Equation (1). The computation requires per-target probabilities over context words, which we calculated based on the raw target–context co-occurrence frequencies.


(1) $$\begin{array}{l} H\left(w\right)=-\underset{c}{\sum }p\left(c|w\right)\cdot log_{2}\left(p\left(c|w\right)\right) \end{array}$$

#### 3.3.3. Weeds Precision

Weeds Precision (WeedsPrec) represents an asymmetric method suggested by Weeds et al. (2004) that quantifies the weighted inclusion of the features of word *w*_1_ in the features of word *w*_2_. In our case the features refer to the words' context words *c*. The underlying assumption is that more context words *c* of the more specific hyponym are among its hypernym's context words than there are context words of the more general hypernym among its hyponym's context words. If *WeedsPrec*(*w*_1_, *w*_2_) > *WeedsPrec*(*w*_2_, *w*_1_), then *w*_1_ is predicted as the hyponym and *w*_2_ as the hypernym, and vice versa, see Equation (2). For example, one would expect more context words of the hyponym *cat* also as context words of its hypernym *animal* (such as *eyes, fur, tail*) than vice versa, because the hypernym also co-occurs with words relevant for other animals (such as *flapper* for *fish*) that are however not relevant for *cats*.

The computation requires raw target–context co-occurrence frequencies |*w*_*ic*_|. Next to the original weighted, token-based version of WeedsPrec in Equation (2) we also apply a non-weighted, type-based version (WeedsPrec′) where we compute *whether* a context word is included in a specific vector, rather than *how often* it is included, see Equation (3).


(2) $$\begin{array}{l} WeedsPrec\left(w_{1},w_{2}\right)=weeds\text{–}token=\frac{\sum_{c\in \left(\vec{w_{1}}\cap \vec{w_{2}}\right)}|w_{1c}|}{\sum_{c\in \vec{w_{1}}}|w_{1c}|} \end{array}$$

(3) $$\begin{array}{l} WeedsPrec^{\prime }\left(w_{1},w_{2}\right)=weeds\text{–}type=\frac{\sum_{c\in \left(\vec{w_{1}}\cap \vec{w_{2}}\right)}1}{\sum_{c\in \vec{w_{1}}}1} \end{array}$$

## 4. Distributional Abstraction Experiments

In this section we report our empirical experiments on distributional models of abstraction. Section 4.1 describes the setup of the experiments, and section 4.2 presents the results of distinguishing between degrees of abstraction in terms of concreteness and hypernymy.

### 4.1. Abstraction Experiments: Setup

#### 4.1.1. Main Experiments

The nature of our target datasets differs with respect to the underlying type of abstraction. For this reason, we defined a common strategy to make the results comparable across datasets: As a major point of comparison we rely on **pairs** of target words, which combine abstract with concrete words, and hypernyms with their hyponyms. For the hypernymy pairs, the two words are directly provided by the resource: we paired each word in a synset with each word in the superordinated synset(s), see section 3.1; for the concrete–abstract pairs, we followed our previous work (Naumann et al., 2018; Frassinelli and Schulte im Walde, 2019) and took our collection of extremes with 500+500 nouns and 200+200 verbs to create 250,000/40,000 concrete–abstract noun/verb word pairs. Note that Figure 1 already included the distributions of concreteness scores for these extreme target subsets.

The task for our measures regarding target pairs was to identify the more abstract word in each pair. The results are computed by determining precision (which in this setup is identical to accuracy), i.e., the proportion of empirically identified abstract words that were indeed the more abstract words in the pairs. We focus on precision here because the differences of our vector spaces regarding the proportions of target words they cover (i.e., their recall) is only marginal. We nevertheless include the numbers of retrieved distinctions per measure and target space in the full results in Appendix 2.

In addition to this first set of experiments where we compared all of our abstraction measures on noun and verb concreteness and hypernymy pairs across vector spaces, we then focused on specific aspects in the experimental paradigm, as follows.

#### 4.1.2. Strength of Abstraction

We hypothesised that the measures are more or less successful with respect to how “different” the concrete and abstract words are in their degrees of concreteness (again, for noun and verb targets), and how “different” the hypernyms and hyponyms are in their degrees of specificity (for nouns only, cf. section 3.1). Similarly to the previous experiments, this setup also relies on concrete–abstract and hyponym–hypernym pairs but the target sets were created in a different way.

For concreteness, we took our full concreteness dataset (see section 3.1) and divided the 5,448/1,280 nouns/verbs (separately for each word class) into five equally-sized subsets, after having sorted them by their concreteness scores. Figure 3 shows the distributions of concreteness scores across the five 20% dataset proportions. Then we created pairs using the targets in subset 1 and the targets in subset 2 (i.e., pairing the 20% most abstract words with each of the targets in the second 20% most abstract words), for each of the targets in subset 1 with each of the targets in subset 3, etc., resulting in a total of 1,187,010 pairs per range combination for nouns, and 65,536 pairs per range combination for verbs. In this way, we compare distinctions for pairs that are more or less similar in their degrees of concreteness, rather than the most extreme subsets. Note, in this respect, that the sizes of the boxes in Figure 3 indicate that we are facing a large number of very concrete nouns, while for verbs the majority is located in the range [2;3].

**Figure 3:**
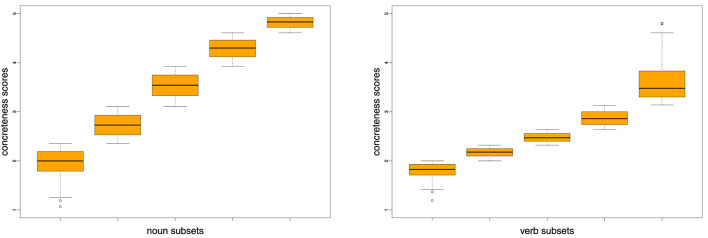
Concreteness ranges of noun and verb subsets (each containing 20% of respective total data).

For hypernymy, we took into account the hierarchical levels of nouns when creating pairs, by pairing the top-level noun in the hierarchy (*entity*) with all second-level nouns, then with all third-level nouns, etc., and by pairing all second-level nouns with all third-level nouns, then with all forth-level nouns, etc. Figure 4 shows the numbers of pairs after combining words from synsets of specific hierarchical hypernymy levels. Note that we go down to level 11 in the WordNet hierarchy for this specific analysis. In the actual experiments we will however disregard the level combinations with <100 pairs (i.e., 1–2, 1–3, 2–3).

**Figure 4:**
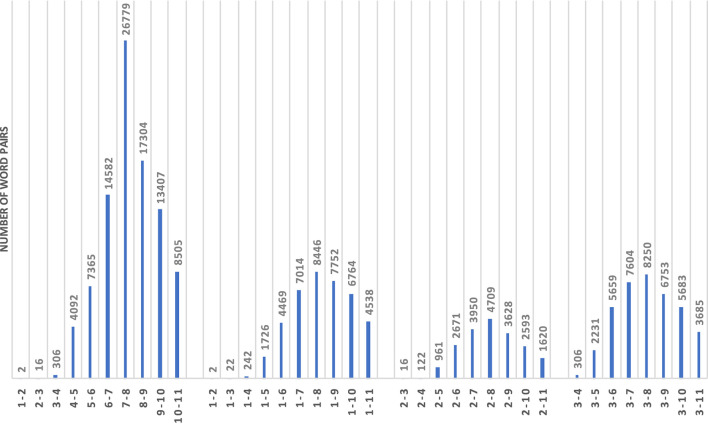
Numbers of word pairs in synset combinations across hierarchical levels.

#### 4.1.3. Correlations and Interactions Between Measures

We zoomed into correlations and interactions of abstraction distinctions across measures, in order to see whether the actual decisions of the measures are more or less strongly correlated with corpus frequency and with each other, and how they interact and complement each other. For this set of experiments we only used the concreteness targets (both nouns and verbs), which provide scores on a scale, differently to the pair-wise organised hierarchical hypernymy targets (which we could organise into hypernymy-based chains of levels but this would add a level of interpretation to the actual human categorisations that we do not judge appropriate). In addition, we used the 329 noun targets from Spreen and Schulz (1966) which are rated on a scale for both concreteness and specificity. For this set of experiments we exploit Spearman's rank-order correlation coefficient ρ (Siegel and Castellan, 1988) and regression models.

We now describe how we apply the abstraction measures to the pair-wise distinction between degrees of abstraction in concrete–abstract pairs and hyponym–hypernym pairs. For measuring contextual word entropy and WeedsPrec, we follow a straightforward one-step procedure: Relying on one of our vector-space matrices, we compute the extent of feature inclusion (WeedsPrec) regarding both words' dimensions, and we compute the word entropy for both words; the comparison of the respective two values then decides which word in a word pair is predicted as the more/less abstract one, see section 3.3. For measuring neighbourhood density, two-step procedures are required: Regarding the CC and TC variants, we first need to identify the *k* strongest context words (i.e., co-occurrence dimensions) for each target word, and then compute the respective average cosine distances between the strongest context words (CC) or between the target and the strongest context words (TC). Regarding the NN and TN variants, we first need to identify the *k* nearest neighbour words for each target word, and then compute the respective average cosine distances between the strongest neighbour words (NN) or between the target and the strongest neighbour words (TN). For all four neighbourhood density variants we rely on one of our vector-space matrices in the first step (i.e., N vs. N-V-A dimensions), and in step two we again face the same choice between the vector-space matrix variants. See Appendix 1 for a selection of noun and verb targets and their strongest context and neighbour words.

### 4.2. Abstraction Experiments: Results

#### 4.2.1. Main Experiments

Figures 5–**8** present the results when distinguishing between degrees of abstraction across measures in terms of precision, i.e., the proportion of abstract words suggested by the measures that were indeed the more abstract words in the pairs. As baseline we use frequency, assuming that a word in a word pair is more abstract if it is more frequent. The weighted vs. non-weighted variants of WeedsPrec are referred to as “weeds-token” vs. “weeds-type,” respectively. For neighbourhood density we report results for 5, 10, 20, and 50 contexts/neighbours across our four variants CC, TC, NN, and TN, and we distinguish between taking into account only nouns or only verbs (depending on the target POS)^3^ as contexts/neighbours vs. *all* nouns, verbs and adjectives (N-V-A). We only show results using the N-V-A vector spaces induced from a co-occurrence window of 20 words, and the density variants that take only single-POS words as contexts/neighbours into account, because these generally provided the best results; the full result tables are available in Appendix 2.

**Figure 5:**
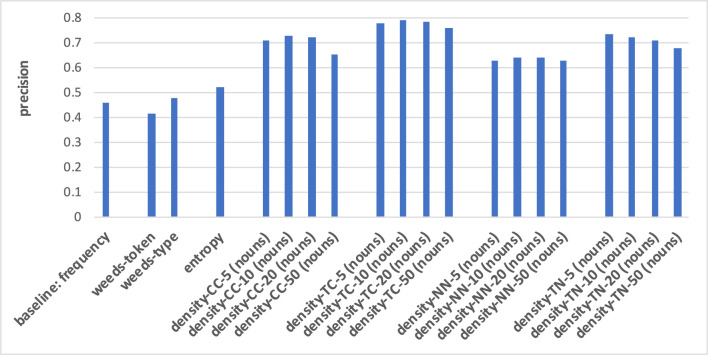
Pair-wise precision results for concreteness of nouns relying on an N-V-A vector space. Densities take only nouns as context/neighbour words into account.

For both noun and verb targets, distinguishing between degrees of concreteness in Figures 5, 6 is performed best when applying the neighbourhood density measure TC: the strength of distributional similarity between a target word and its strongest context words distinguishes between the most abstract and the most concrete words with a precision of up to 0.79 for nouns and 0.67 for verbs, respectively. This means that the distributionally most similar context words in relation to a target are most indicative of the target's concreteness, and the higher this average vector-space similarity is, the more concrete are the target words. The next-best variants differ across the two POS types of our targets: for noun targets, the density measures are generally better than the baseline, weeds-token/-type and entropy, with density-NN representing the worst of the four density variants; for verb targets, the other density variants are at most en par with the baseline, weeds-token/-type and entropy, and overall the density variants are worse than for nouns, while the other measures perform better distinctions than for nouns. I.e., the baseline, weeds-token and entropy achieve 0.46/0.42/0.53 for nouns and 0.54/0.54/0.57 for verbs; for nouns the frequency baseline is even below the random baseline of 0.5. An additional insight from the figures is that in the vast majority of cases the strongest five or ten contexts/neighbours are the most indicative of their degrees of concreteness: in most cases the results worsen when more contexts/neighbours are taken into account. Including as contexts/neighbours only nouns/same-POS words (as in Figures 5, 6, cf. footnote 3) vs. nouns, verbs and adjectives (see “all” in the full result tables in the Appendix) does not seem to strongly influence the qualities of the distinctions.

**Figure 6:**
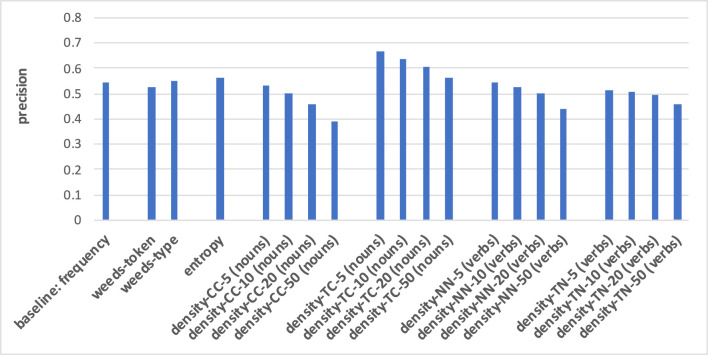
Pair-wise precision results for concreteness of verbs relying on an N-V-A vector space. Densities take only nouns as context/neighbour words into account.

The prediction of hypernymy in Figures 7, 8 provides a totally different pattern of results. For both noun and verb targets the best results are achieved by the frequency baseline (0.73/0.71), entropy (0.72/0.71), and the WeedsPrec variants: 0.72/0.73 for weeds-token and 0.73/0.71 for weeds-type, in comparison to the best density variants (for noun targets and density-NN-5: 0.52; for verb targets and density-NN-10: 0.56). Overall, most of the density-based results hardly beat the random baseline (0.5). Furthermore, the tendency that the density-based distinction results decrease when taking more context/neighbour words into account is visible only in some variants, and also not as clearly as in the results for distinguishing between degrees of concreteness.

**Figure 7:**
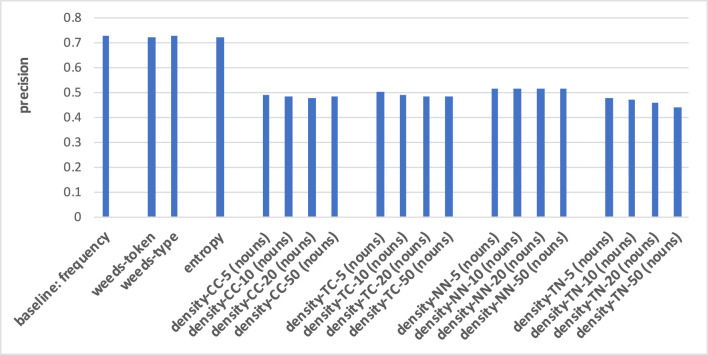
Pair-wise precision results for hypernymy of nouns relying on an N-V-A vector space. Densities take only nouns as context/neighbour words into account.

**Figure 8:**
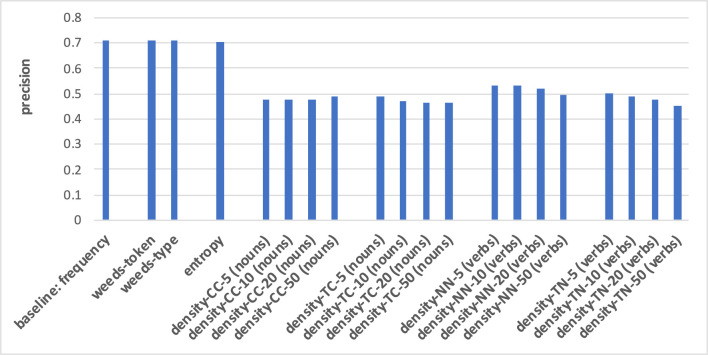
Pair-wise precision results for hypernymy of verbs relying on an N-V-A vector space. Densities take only nouns as context/neighbour words into account.

#### 4.2.2. Strength of Abstraction

Following the main set of experiments we now zoom into the role of differences in results according to the strengths of concreteness and the levels of hypernymy. We hypothesise that the measures are more or less successful with respect to how “different” the concrete and abstract words are in their degrees of concreteness, and how “different” the hypernyms and hyponyms are in their degrees of specificity. We once more compare the baseline, weeds-token/-type, and entropy; for the neighbourhood variants we present the results relying on the 10 strongest context/neighbour words, because these proved rather successful and stable in the main experiments, and here we are not interested in the best results but rather in tendencies across subsets.

Figure 9 shows the results^4^ across four sets of combinations of concreteness degrees for nouns. Note that we use the interval [0.4;0.8] for precision values on the y-axis, for better visibility of trends and differences in results. The left-most set of results compares the distinctions between the most abstract and the second most abstract 20% of the targets, then the second and the third most abstract 20% of the targets, etc. So in this first set, the distances between concreteness degrees are identical (i.e., we use adjacent levels), but the concreteness ranges of the involved subsets differ. We can see that for the best three measures (densities TC, CC and TN) there is a slight upward trend which only drops for a mid-range comparison (subsets 3–4), even though we always look at adjacent levels. The four measures frequency, entropy and weeds-token/-type are better for mid-range nouns than for extremely abstract/concrete nouns but overall obtain lower precision values than the above three density variants. Density-NN shows the most idiosyncratic pattern of results, with mid-range precision values.

**Figure 9:**
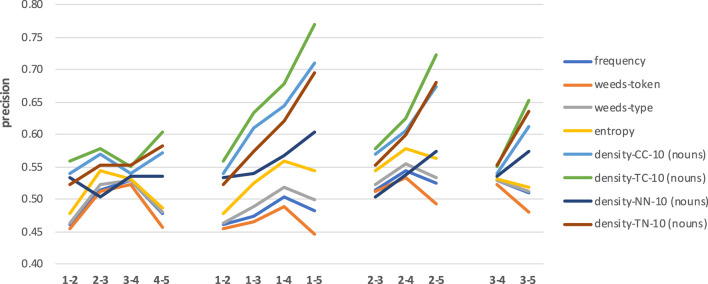
Results across combinations of concreteness ranges for nouns.

When comparing the results for nouns with increasing differences in concreteness degrees (see second, third and forth sets of results, using reference labels 1, 2, and 3), we can clearly see that for the four density variants the task becomes easier (and, accordingly, the results of the best measures improve) with stronger differences in concreteness scores. The overall best result (0.77) is obtained when distinguishing between nouns in levels 1 vs. 5, which represents the strongest difference in concreteness scores and is therefore similar to the previous extreme-range distinctions in the main experiments. The measures frequency, entropy and weeds-token/-type also show a slight increase in precision values but then drop for every comparison involving the most extreme concrete nouns (i.e., set 5).

Regarding abstraction measures, our insights from the main experiments are confirmed: for distinguishing between degrees of noun concreteness, the neighbourhood density measure TC is the best and most consistent in all cases, density-TN and density-CC are the next-best measures, and density-NN as well as frequency, entropy and weeds-token/-type represent the least successful measures.

Figure 10 shows the results across four sets of combinations of concreteness degrees for verbs. Note that we now use the interval [0.4;0.65] for precision values on the y-axis, for better visibility of trends and differences in results. The left-most set of results across concreteness ranges for adjacent subsets shows a less clear pattern than for nouns. Across measures, the best results are achieved for the most abstract and for the most concrete subset combinations (1–2 and 4–5) and drop for the middle range combinations (2–3 and 3–4).

**Figure 10:**
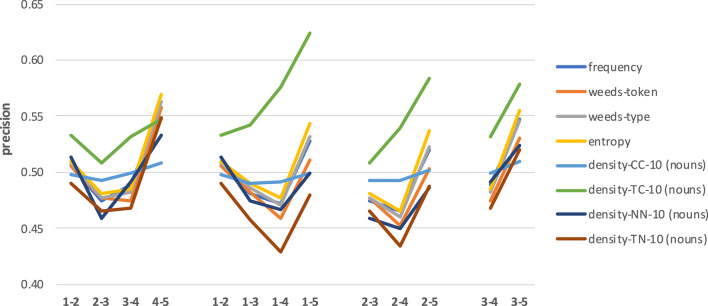
Results across combinations of concreteness ranges for verbs.

When comparing the results for verbs with increasing differences in concreteness degrees (see second, third and forth sets of results, again using reference labels 1, 2, and 3), we can see that the task is once more the easiest for the strongest differences in concreteness scores. But as for the adjacent-level comparisons for verb subsets, decisions involving the middle ranges are worse. Overall, the results are clearly below those for nouns, with a best result of 0.62 obtained by density-TC when distinguishing between verbs in levels 1 vs. 5.

Regarding abstraction measures, our insights from the main experiments are confirmed to some extent: for distinguishing between degrees of verb concreteness, the neighbourhood measure density-TC is the best in most cases, and frequency, entropy and weeds-token/-type are extremely similar to each other and represent the next-best set of measures, however clearly below density-TC precision results and not much above the other density variants. Density-CC seems to be least influenced by the degree of concreteness, showing similar results across comparisons.

Figure 11 shows the results across four sets of combinations of hypernymy levels for nouns. Note that in this case we use the full interval [0;1] for precision values on the y-axis. The left-most set of results compares the distinctions between pairs of related nouns from adjacent levels of hypernymy. Please remember that we omit the combinations 1–2, 1–3, and 2–3 because these sets of pairs contain only 2, 16, and 22 pairs, respectively. Differently to the noun concreteness distinctions, there seems to be a slight downward trend in precision. At the same time, there is more up and down across the level combinations, so the trends are also less clear overall. What is clearly visible, on the contrary, is that frequency, entropy and weeds-token/type are by far the best measures in this left-most set of distinctions for directly hypernymy-related nouns across levels in the hierarchy (down to level 11).

**Figure 11:**
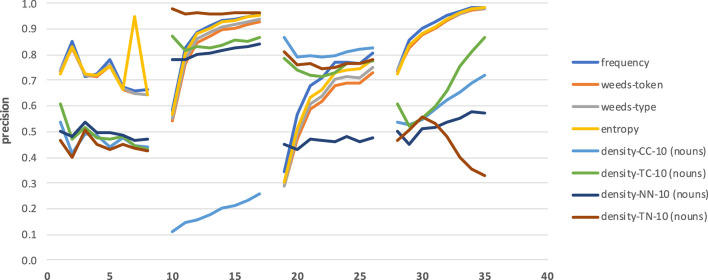
Results across combinations of hypernymy levels for nouns.

Similarly, when comparing the results for related nouns with increasing differences in hypernymy levels (see second, third and forth sets of results, again using reference levels 1, 2, and 3), we can clearly see that also here the task becomes easier (and, accordingly, the results improve) with stronger differences in hypernymy levels. While this is clearly true for frequency, entropy and weeds-token/type, the patterns differ more strongly for the density variants which mostly show less variability in results. Similarly to the main results for hypernymy prediction, we once more observe that frequency, entropy and weeds-token/-type generally represent the best measures, while the density variants are worse.

#### 4.2.3. Correlations and Interactions Between Measures

Overall, when looking at the distributions of frequency, entropy, weeds-token/-type and the neighbourhood densities across types of abstraction and POS we see how subgroups of the measures are often extremely similar to each other (and possibly interchangeable) in terms of predictive power. We now zoom into correlations and interactions of abstractness distinctions across abstraction measures, in order to see whether the actual scores provided by the measures are more or less strongly correlated with corpus frequency and with each other, and how they interact and complement each other. For this set of experiments we thus compare scores for words rather than binary decisions for word pairs, and as mentioned above we use our concreteness targets (both nouns and verbs), which provide scores on a scale, and we use the 329 noun targets from Spreen and Schulz (1966) because those were rated on a scale for both concreteness and specificity. We disregard the weeds-token-type precision measures, as they would require setting additional parameters in order to generate one score out of the two scores per pair.

##### 4.2.3.1. Correlations

Figure 12 shows the correlations between noun concreteness scores, corpus frequency, entropy, and our four neighbourhood density variants (once more relying on k=10). As before, the measures use N-V-A spaces with a window of 20 words. First of all, we can see that the concreteness scores using entropy are strongly correlated with corpus frequency (ρ=0.964), while the density measures show no or very low correlations with corpus frequency and entropy, so the density measures produce rather different scores for abstraction in comparison to frequency and entropy. Among themselves, the density measures show stronger agreement on their scores: regarding context densities, CC-10 and TC-10 correlate strongly (ρ=0.814); regarding nearest neighbour densities, NN-10 and TN-10, we find ρ=0.719. In contrast, we see low correlations for NN-10 with CC-10/TC-10 (ρ<0.3), while for TN-10 we find medium-level correlations of ρ≈0.5 with the two context variants.

**Figure 12:**
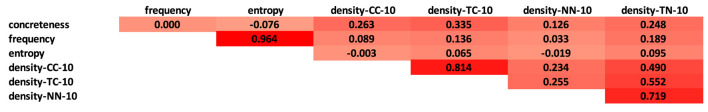
Spearman's ρ correlations between noun concreteness measures (N-V-A space).

Figure 13 shows the correlations between verb concreteness scores, corpus frequency, entropy and our four neighbourhood density variants (k=10). As for the nouns, we find extremely high correlations between corpus frequency and entropy; no correlations between these two measures and concreteness scores; strong correlations for CC-10/TC-10 and NN-10/TN-10; moderate correlations between TN-10 and the context variants; and low correlations between NN-10 and the context variants. Differently to the noun distinctions, we do not find any correlation between any of the abstraction measures and concreteness.

**Figure 13:**
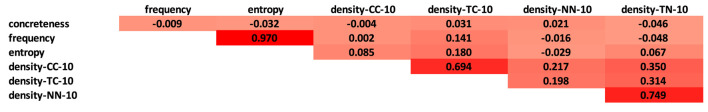
Spearman's ρ correlations between verb concreteness measures (N-V-A space).

Figures 14, 15 look into correlations between abstraction ratings and abstraction measures for a subset of 226 noun targets from Spreen and Schulz (1966). These 226 targets represent the intersection of the nouns in Spreen and Schulz (1966) and our full concreteness subset Brysbaert et al. (2014). First of all, Figure 14 shows the correlations between the concreteness and specificity ratings for these 226 noun targets in the two norms. The two sets of concreteness ratings, which represent the main point of comparison, strongly correlate (ρ=0.939). Between the two sets of concreteness ratings and the specificity ratings we find a lower but still meaningful correlation of ρ≈0.7 for both resources (Note that Spreen and Schulz (1966) report a correlation of 0.626 between the concreteness and specificity ratings for their full set of 329 nouns).

**Figure 14:**
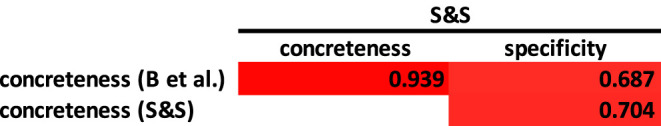
Spearman's ρ correlations between the Spreen and Schulz (1966) and Brysbaert et al. (2014) ratings for the subset of 226 nouns in the intersection.

**Figure 15:**
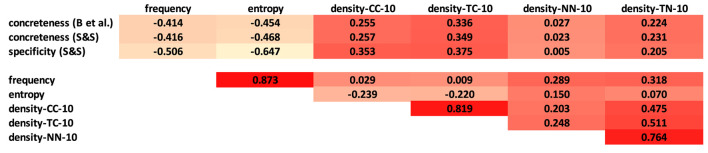
Spearman's ρ correlations between ratings and measures for the subset of 226 nouns in the intersection of Spreen and Schulz (1966) and Brysbaert et al. (2014).

As in Figures 12, 15 shows the correlations between noun concreteness scores, corpus frequency, entropy, and our four neighbourhood density variants (once more relying on k=10) for the set of 226 nouns, once more using N-V-A spaces with a window of 20 words. The overall picture is very much the same as for our full set of 5,448 target nouns in Figure 12, for the concreteness ratings in Brysbaert et al. (2014) and the concreteness and specificity ratings in Spreen and Schulz (1966), with one exception: frequency and entropy show a moderate negative correlation with all abstraction rating sets: −0.47<ρ<−0.41 for both sets of concreteness ratings, and −0.65<ρ<−0.51 for specificity ratings. The outcome of this last analysis is in line with what we would have expected (but did not happen) to see in all three figures: generally, abstract nouns are more frequent/entropic than concrete nouns, as we will also see below in the regression analysis, so we expected a negative correlation between both frequency and entropy and the concreteness ratings.

Overall, the correlations for nouns and verbs (and for our targets and the subset of the targets from Spreen and Schulz, 1966) show similar patterns regarding strong frequency–entropy correlations and tendencies in the intra- and extra-density correlations. We however did not observe any meaningful correlation between the abstraction measures and the concreteness scores of our verb targets, while we found correlations of ρ≈0.3 between the abstraction measures and our noun ratings. This fits to our insights from the main experiments, where the pair-wise distinctions for concreteness of verbs were worse than for nouns, and often similar to a random baseline; nevertheless we reached precision scores of up to 0.79/0.67 for nouns/verbs, respectively. For the much smaller set of 226 nouns from Spreen and Schulz (1966) the picture is similar to that for our noun targets, but in addition frequency and entropy show a moderate negative correlation with both concreteness and specificity ratings.

##### 4.2.3.2. Interactions

The correlation analysis reported in Figure 12 shows a strong positive relationship for nouns in the N-V-A space between frequency and entropy as well as between the density variants TC, CC, TN, and NN. For this reason, we must consider collinearity issues between the various predictors (features) when modeling concreteness using linear regression models. In the following analyses, we will model concreteness (as a continuous value ranging from 1 to 5) given different feature combinations. After centering around the mean all the predictors, to test which triplet of variables best captures variability in concreteness scores, we run eight independent models and select the one with the highest adjusted R-squared value, as a measure of explained variance in the data. For an overview of the performance of the eight models, see Table 3. The model including entropy, density-TC, and density-TN (highlighted by bold font) is the one explaining the highest amount of variance in the concreteness scores (adjusted R-squared: 13.4%) and does not show any collinearity problem (VIF<1.64). For this reason, we will focus the following analysis on this model. The results discussed below are also fully in line with the results in the other seven models from Table 3. As shown in Table 4, all three predictors (entropy, density-TC, density-TN) are highly significant (*p*<0.0001, after alpha correction because of multi-comparisons) when modeling the concreteness of a noun. Words that are more concrete show: significantly lower entropy scores, higher density-TC and higher density-TN; moreover, the interaction between the two density measures indicates a positive overall effect. In the same table, we also report the “relative importance” of each predictor (normalised to 100%) using the method developed by Lindeman et al. (1980). This measure indicates the contribution of each predictor to the total amount of variance explained by the model. Density-TC by itself explains 68.7% of the variance captured by the model, density-TN 20.7% and entropy only 7.3%. The contribution of the various features is very stable across models and in line with what has been discussed in the previous sections. When looking at all eight models, density measures involving contextual information like density-TC and density-CC always contribute the most, as opposed to nearest neighbour measures like density-NN and density-TN.

**Table 3:** Comparison of model variants processing noun targets in the N-V-A space, and their explained variance (represented in terms of adjusted R-squared).

**Formula**	**Adj. R-squared (%)**
freq (ENCOW) + (density-TC × density-TN)	12.5
freq (ENCOW) + (density-TC × density-NN)	11.9
freq (ENCOW) + (density-CC × density-TN)	9.3
freq (ENCOW) + (density-CC × density-NN)	8.1
**entropy + (density-TC × density-TN)**	**13.4%**
entropy + (density-TC × density-NN)	12.8
entropy + (density-CC × density-TN)	9.9
entropy + (density-CC × density-NN)	8.5

*The dependent variable is concreteness (1–5)*.

**Table 4:** Linear regression output for the best predictor combination for nouns in the N-V-A condition: entropy + (density-TC × density-TN).

	**Estimate**	**Std. error**	***t*-value**	***p*-value**	**RI (%)**
*(Intercept)*	3.44	0.01	234.91	***	-
entropy	−0.11	0.01	−8.53	***	7.3
density-TC	2.80	0.17	16.76	***	68.8
density-TN	0.83	0.12	7.07	***	20.7
density-TC × density-TN	4.45	0.86	5.20	***	2.3

*RI indicates the relative importance (normalised to 100%)*.

*The significance codes are all adjusted because of the 8 multi-comparisons*.

Significance codes: *p<0.006, **p<0.001, ***p<0.0001.

In Table 5, we see similar patterns to those emerged for nouns also for verbs. Once again, the model including entropy, density-TC and density-TN is the one obtaining the highest R-squared value. However, compared to nouns, the explained variance is extremely low (only 2%). When zooming in on the effect of the single predictors on concreteness, Table 6 indicates some differences. The model shows only a strong significant positive effect of density-TC (*p*<0.0001; after alpha correction) indicating that the contextual density of concrete words is higher than the abstract one. For verbs, entropy (*p*=0.008), density-TN (*p*=0.031) and the interaction between the two density measures (*p*=0.910) do not reach significance. Once more, density-TC is the feature with the strongest effect on concreteness scores, both for nouns and verbs.

**Table 5:** Comparison of model variants processing verb targets in the N-V-A space, and their explained variance (represented in terms of adjusted R-squared).

**Formula**	**Adj. R-squared (%)**
freq (ENCOW) + (density-TC × density-TN)	1.5
freq (ENCOW) + (density-TC × density-NN)	1.2
freq (ENCOW) + (density-CC × density-TN)	−0.2
freq (ENCOW) + (density-CC × density-NN)	−0.2
**entropy + (density-TC × density-TN)**	**2.0**
entropy + (density-TC × density-NN)	1.6
entropy + (density-CC × density-TN)	0.0
entropy + (density-CC × density-NN)	0.0

*The dependent variable is concreteness (1–5)*.

**Table 6:** Linear regression output for the best predictor combination for verbs in the N-V-A condition: entropy + (density-TC × density-TN).

	**Estimate**	**Std. error**	***t*-value**	***p*-value**	**RI (%)**
*(Intercept)*	2.58	0.02	140.42	***	-
entropy	−0.04	0.02	−2.67		18.5
density-TC	1.21	0.25	4.84	***	72.4
density-TN	−0.33	0.15	−2.16		9.0
density-TC × density-TN	−0.16	1.40	−0.11		0.0

*RI indicates the relative importance (normalised to 100%). The significance codes are all adjusted because of the 8 multi-comparisons*.

Significance codes: *p<0.006, **p<0.001, ***p<0.0001.

## 5. Discussion

The previous section provided a series of vector-space experiments to investigate two conceptual categorisations of lexical-semantic abstraction (abstractness–concreteness and generality–specificity) through variants of distributional computational measures. The current section summarises, interprets and discusses the insights from the empirical experiments with respect to differences in the conceptual organisation of English nouns and verbs, and the roles of corpus frequency, distributional co-occurrence, distributional similarity and distributional neighbourhoods for mental distinctions between degrees of semantic abstraction.

Our experiments brought together a variety of distributional vector-space measures that had previously been applied to different tasks of lexical-semantic abstraction. We focused on the two types of semantic abstraction originally suggested by Spreen and Schulz (1966) and brought back to attention by Theijssen et al. (2011) and Bolognesi et al. (2020). They distinguished abstraction in terms of the abstract–concrete dichotomy (e.g., *glory* is more abstract than *banana*), and abstraction in terms of the generality–specificity distinction (e.g., *animal* is more abstract than *fish*). Assuming that a large-scale web corpus provides an adequate basis for general-language distributional information, we empirically explored corpus frequency and corpus co-occurrence as proxies to lexical-semantic meaning and lexical meaning relatedness. We thereby relied on the distributional hypothesis (Harris, 1954; Firth, 1957) indicating that words which are similar in meaning also occur in similar linguistic distributions.

In this vein, we induced variants of neighbourhood densities (context-based and neighbour-based), token- and type variants of the distributional, vector-based inclusion measure *WeedsPrec*, as well as word frequency and word entropy, in order to empirically capture noun and verb target words differing in their degrees of semantic abstraction. We applied these distributional measures to distinguish between degrees of abstraction regarding the abstract–concrete dichotomy as well as regarding the generality–specificity distinction. Overall, we identified reliable vector-space measures for both instantiations of lexical-semantic abstraction (reaching a precision higher than 0.7), but the measures clearly differed for concreteness vs. hypernymy and for nouns vs. verbs. In order to distinguish between more and less abstract words in terms of hypernymy, we found that word frequency computed on corpus data, word entropy, and the distributional inclusion measure (originally suggested for hypernymy) were the most salient predictors, while neighbourhood density measures could hardly beat the random baseline. In order to distinguish between more and less abstract words in terms of concreteness, the neighbourhood density measures were generally more successful than frequency, word entropy and distributional inclusion, especially when integrating only the strongest contexts/neighbours. Among the density measures the variant that considers the distributional similarity between a target word and its strongest context words (density-TC) seems the most appropriate and is also the one with the highest impact in the regression studies. This overall picture was similar for concreteness ratings for nouns and verbs, but (i) the precision scores for verbs were generally lower than for nouns and could hardly beat the random baseline, and (ii) frequency, entropy and weeds-token were not much different from (or even better than) the density variants CC, NN and TN.

As a side line of research we explored differences in distinctions between degrees of abstraction regarding variants of vector spaces in the experimental paradigm. While our main set of experiments did not go into depth regarding this variable, our full results in the Appendix demonstrate surprisingly clear differences regarding window size and parts-of-speech of vector dimensions: Results exploiting vector spaces induced from a co-occurrence window of ±20 words (in comparison to only ±2 words) and density variants taking only single-POS words as contexts/neighbours into account generally provided the best results. Whether it was more profitable to rely on noun-only vs. N-V-A (nouns, verbs, adjectives) dimensions in the co-occurrence vectors depended on the target POS and type of abstraction: For noun concreteness the N-V-A spaces seemed more indicative, while for verb concreteness and noun and verb specificity the noun-only spaces were more salient.

When zooming into the role of measure-based distinctions according to the strengths of concreteness and the levels of hypernymy, i.e., hypothesising that the measures are more or less successful with respect to how “different” the concrete and abstract words are in their degrees of concreteness, and how “different” the hypernyms and hyponyms are in their degrees of specificity, our insights from the main experiments were largely confirmed and partially even strengthened: The stronger the differences in concreteness, the better the quality of distinctions in terms of precision. While this is true for both noun and verb targets, the picture was again clearer for nouns than for verbs; in the latter case, distinctions for target verbs involving the mid-range scale of concreteness were worse than those involving any of the extreme ranges. Taking into account that the concreteness ranges for verbs in the mid-range subsets are rather small ([2.0;2.3] for subset 2; [2.3;2.6] for subset 3; and [2.6;3.1] for subset 4), this tendency is reasonable because concreteness scores from different subsets were still rather similar to each other. Also, mid-range concreteness scores are generally more difficult in their generation by humans and consequently noisier in their distributional representation (Pollock, 2018). Finally, verbs are generally more ambiguous than nouns, especially when their semantic properties have been evaluated out of context, and furthermore perception-based concreteness ratings might not be as appropriate for verbs as they are for nouns. Regarding abstraction measures, our zooming-in experiments confirmed that the target–context measure density-TC is the best one for predicting abstraction in terms of concreteness, while frequency, entropy and weeds-token/-type are the best ones for predicting abstraction in terms of hypernymy.

A final study looked into correlations between concreteness and specificity ratings, the abstraction measure, and their interactions. These correlations confirmed that corpus frequency and word entropy measure abstraction in a similar way, and ditto for the context-based density measures CC and TC and the neighbour-based density measures NN and TN (while density-NN seems to differ most from the other density variants). Moreover, based on a series of regression studies, we confirmed that density-TC is the strongest option to quantify concreteness both for nouns and for verbs.

Bringing together our results across experiments, we can identify two groups of measures, (i) frequency and word entropy, whose distinctions are correlated and which are stronger than neighbourhood density measures when distinguishing between more and less abstract words in terms of the generality–specificity distinction, and (ii) the neighbourhood density variants, which are stronger than group (i) when distinguishing between more and less abstract words in terms of the abstractness–concreteness dichotomy. The distributional inclusion variants of WeedsPrec cluster together with frequency and entropy, and are clearly more useful for hypernymy than for concreteness. Regarding group (i), the relationship between frequency, word entropy and the lexical-semantic relation hypernymy has been demonstrated before (Shwartz et al., 2017; Bott et al., 2021), and our experiments confirmed this strong interaction across a variety of experimental conditions regarding strength of hypernymy. Regarding group (ii), we effectively and successfully exploited the usefulness of neighbourhood density measures that had previously been suggested and applied to different instantiations of lexical-semantic abstraction. At the same time we demonstrated that there are indeed conceptual differences between the measures that result in different distinction qualities for our two target types of abstraction.

Now let us look at these empirical results and insights from a conceptual perspective. First of all, we can induce from our results that lexical-semantic abstraction in terms of generality in the human lexicon is mirrored by how often we use words, which itself is highly correlated with the words' entropy values. While this is neither surprising nor novel, one might not have expected such a clear picture over diverse settings regarding degrees of generality. That is, more general words are used more often and are therefore also less surprising. The density measures do not seem appropriate to model the generality–specificity distinction, thus indicating that they do not capture degrees of semantic relatedness (which is taken into account by the vector similarity variants of WeedsPrec, for example). Secondly, we can induce from our results that contextual diversity/neighbourhood density is a strong indicator of lexical-semantic abstraction in terms of concreteness. Given that density-TC seems to represent the overall most salient measure, we may induce that abstract words establish themselves empirically in semantically more diverse contexts than concrete words, thus abstract concepts are lexically connected to more different concepts, while concrete concepts are lexically connected to less diverse but on the other hand semantically more strongly associated concepts, and these semantically most indicative associated words are predominantly represented by nouns. In this vein, lexical entries of abstract and concrete words may be refined with respect to their tendencies to co-occur with more or less highly distributionally similar, and consequently—according to the distributional hypothesis—also more or less semantically related words (nouns). The differences in the success of the abstraction measures regarding our two target types of semantic abstraction seems directly related to a core distinction: while words differing in their degree of concreteness are not necessarily semantically related (e.g., *glory–banana*), words differing in their degree of specificity (e.g., *animal–fish*) are, at least with regard to hypernymy in WordNet. Overall, our insights should generally be useful for computational models exploiting degrees of semantic abstraction, such as standard classification approaches and topic models, and similarly for more complex computational systems where the degree of contextual abstraction plays a role, such as figurative language detection, text simplification, summarisation, and machine translation.

Our experiments also point out once more that distributional measures, distributional similarity and distributional semantic relatedness differ across word classes. On the one hand, concreteness and hypernymy represent two lexical-semantic types of abstraction, and therefore their organisation is also defined in different ways in the respective resources. That is, concreteness scores had been collected on a word-type basis, where participants were not provided a part-of-speech categorisation and part-of-speech tags were assigned *post-hoc*. Even though we applied a rather restrictive procedure to POS label identification and discarded ambiguous words, this basis is sub-optimal for any word-class-dependent analyses: we calculated Spearman's ρ correlation for the POS assignment based on SUBTLEX (Brysbaert et al., 2012) and our ENCOW-based procedure, obtaining ρ=0.624 for our noun targets and ρ=0.750 for our verb targets, which we consider as rather low and pointing to an undesired disagreement in POS assignment. On the other hand, all our studies have been on a type-basis: vector spaces and concreteness ratings are type-based, and while WordNet does distinguish between word senses, we only indirectly used this option, because we utilised all senses in word pairs, but we did not distinguish between senses. This is more crucial for verbs than for nouns, which are notoriously more ambiguous. Overall, future work should therefore target contextualised, token-based distributional representations and sense-based abstraction ratings.

## 6. Conclusion

In this article, we provided a series of empirical studies that investigated conceptual categories of semantic abstraction through distributional variants of abstraction measures. We distinguished abstraction in terms of the abstract–concrete dichotomy and in terms of the generality–specificity distinction, and brought together a variety of distributional measures that had previously been applied to different tasks of lexical-semantic abstraction. We thus suggested a novel perspective that exploited empirical measures across two types of semantic abstraction, in order to compare the strengths and weaknesses of the measures for categorisations of abstraction, and to determine and investigate conceptual differences as captured by the measures.

In a series of experiments we identified reliable vector-space measures for both instantiations of lexical-semantic abstraction (reaching a precision of >0.7), and we demonstrated that the measures clearly differed for concreteness vs. hypernymy and for nouns vs. verbs. We could identify two groups of measures, (i) frequency, word entropy and weeds-token/-type when distinguishing between more and less abstract words in terms of the generality–specificity distinction, and (ii) the neighbourhood density variants (especially target–context diversity, with nouns providing the most salient context words) when distinguishing between more and less abstract words in terms of the abstractness–concreteness dichotomy. We concluded that more general words are used more often and are therefore also less surprising than more specific words, and that abstract words establish themselves empirically in semantically more diverse contexts than concrete words, i.e., abstract concepts are lexically connected to more different concepts, while concrete concepts are lexically connected to less diverse but at the same time semantically more strongly associated concepts.

Finally, we demonstrated the need to take word classes and ambiguity into account. On the one hand, results for nouns vs. verbs clearly differ, and both ratings and vector spaces should take semantic differences between word classes into account; on the other hand, ambiguity (which is more severe for verbs than for nouns) prevents from fine-tuning empirical observations and conclusions.

## Data Availability Statement

The original contributions presented in the study are included in the article/Supplementary Material, further inquiries can be directed to the corresponding author/s.

## Author Contributions

All authors listed have made a substantial, direct, and intellectual contribution to the work and approved it for publication.

## Conflict of Interest

The authors declare that the research was conducted in the absence of any commercial or financial relationships that could be construed as a potential conflict of interest.

## Publisher's Note

All claims expressed in this article are solely those of the authors and do not necessarily represent those of their affiliated organizations, or those of the publisher, the editors and the reviewers. Any product that may be evaluated in this article, or claim that may be made by its manufacturer, is not guaranteed or endorsed by the publisher.

## Footnotes

^1^https://www.webcorpora.org/encow/ provides details on corpus version and toolchains.

^2^See http://www.collocations.de/AM/ for a detailed description of association measures.

^3^When taking into account a single POS for context/neighbour words, as context words we use nouns for both noun and verb targets, and as nearest neighbours we use same-POS neighbour words (i.e., noun nearest neighbours for noun targets and verb nearest neighbours for verb targets).

^4^Note that even though the precision scores are discrete, we use lines to illustrate the results, for better visibility and comparison.
