# Supplementary material for: Distributional Measures of Semantic Abstraction
Source: Front Artif Intell. 2022 Feb 8;4:796756. doi: 10.3389/frai.2021.796756 (PMC8892386; doi:10.3389/frai.2021.796756)
Supplement: Supplementary file 1 [file Presentation_1.pdf]

1 EXAMPLES: CONTEXT AND NEIGHBOUR WORDS

Table 1 shows the five strongest context and neighbour words for a small subset of noun and verb targets, in order to get an impression of conceptual differences between context and neighbour words. Note that strongest noun context words as used in the density-CC and density-TC variants have been selected based on target–context plmi scores, and that strongest nearest neighbours as used in the density-NN and density-TN variants have been selected based on target–neighbour cosine scores, here showing the respective noun neighbours for noun targets and verb neighbours for verb targets.

targets		mean ratings	strongest contexts			strongest neighbours	
			word	plmi	cosine	word	cosine
N concrete	wine	4.79	bottle	174,811	0.75	vino	0.83
			glass	158,713	0.63	demijohn	0.81
			beer	92,498	0.58	rosé	0.81
			grape	69,048	0.65	sommelier	0.79
			food	55,781	0.18	tasting	0.79
	trout	4.72	fishing	45,436	0.55	grayling	0.81
			salmon	31,941	0.70	steelhead	0.77
			rainbow	28,065	0.62	salmon	0.70
			fish	19,793	0.38	whitefish	0.68
			lake	14,159	0.41	kokanee	0.65
N abstract	wisdom	1.53	knowledge	33,767	0.29	fount	0.51
			word	21,322	0.20	foolishness	0.47
			man	14,678	0.11	prajna	0.46
			love	12,914	0.20	sagacity	0.43
			power	10,539	0.12	folly	0.41
	sensibility	1.52	sense	6,559	0.12	aesthetic	0.43
			film	2,520	0.19	humor	0,42
			pop	2,347	0.19	expansiveness	0.38
			sensuality	2,277	0.32	rootlessness	0.38
			art	1,589	0.21	purposefulness	0.38
V concrete	sit	4.80	room	152,949	0.46	seat	0.68
			table	144,106	0.57	plop	0.61
			chair	134,806	0.63	scoot	0.61
			front	75,121	0.42	slouch	0.51
			seat	71,815	0.30	plonk	0.50
	breathe	4.07	air	64,932	0.51	humidify	0.59
			sigh	38,937	0.42	dehumidify	0.58
			life	27,472	0.25	condition	0.56
			relief	24,369	0.29	rarefy	0.55
			breath	23,892	0.41	gasp	0.54
V abstract	expect	1.89	result	29,932	0.22	anticipate	0.60
			price	29,408	0.35	forecast	0.43
			week	28,254	0.26	come	0.38
			level	28,192	0.28	rise	0.37
			month	27,556	0.33	disappoint	0.36
	overrate	1.86	player	1,529	0.33	underrate	0.73
			film	616	0.14	cogitate	0.70
			opinion	521	0.12	crystalize	0.70
			game	448	0.18	mistake	0.61
			bit	394	0.19	delude	0.59

Table 1. Strongest context and neighbour words for a selection of target nouns and verbs.

2 FULL TABLES OF RESULTS

Tables 2–5 provide the full results for pair-wise distinctions between degrees of abstraction in terms of concreteness and hypernymy, both for nouns and for verbs. We applied symmetric co-occurrence windows of ±2 and ±20 words; vector spaces including only co-occurring nouns (space: N) vs. nouns/verbs/adjectives (space: N-V-A); and density variants taking only nouns or verbs or nouns/verbs/adjectives (all) as context/neighbour words into account. The results show precision scores in combination with the number of pairs for which the distinctions were made. The best result per package is highlighted.

	window 2				window 20			
	space: N		space: N-V-A		space: N		space: N-V-A	
baseline: frequency	0.4574 (250,000)							
weeds-token	0.3797	(166,457)	0.4263	(245,173)	0.3642	(250,000)	0.4157	(250.000)
weeds-type	0.4243	(166,457)	0.4330	(245,173)	0.4673	(250,000)	0.4758	(250,000)
entropy	0.4451	(249,000)	0.4355	(250,000)	0.5255	(250,000)	0.5230	(250,000)
density-CC-5 (nouns)	0.6833	(247,000)	0.6663	(247,000)	0.6965	(250,000)	0.7087	(250,000)
density-CC-5 (all)	0.6513	(250,000)	0.6567	(250,000)	0.6798	(250,000)	0.7044	(250,000)
density-CC-10 (nouns)	0.6863	(247,000)	0.6707	(247,000)	0.7142	(250,000)	0.7272	(250,000)
density-CC-10 (all)	0.6524	(250,000)	0.6554	(250,000)	0.6900	(250,000)	0.7150	(250,000)
density-CC-20 (nouns)	0.6878	(247,000)	0.6505	(247,000)	0.7088	(250,000)	0.7244	(250,000)
density-CC-20 (all)	0.6257	(250,000)	0.6417	(250,000)	0.6648	(250,000)	0.6979	(250,000)
density-CC-50 (nouns)	0.6479	(247,000)	0.5673	(247,000)	0.6395	(250,000)	0.6547	(250,000)
density-CC-50 (all)	0.5647	(250,000)	0.5823	(250,000)	0.5784	(250,000)	0.6233	(250,000)
density-TC-5 (nouns)	0.5713	(248,000)	0.5882	(249,000)	0.7068	(250,000)	0.7799	(250,000)
density-TC-5 (all)	0.6037	(248,500)	0.6475	(250,000)	0.7357	(250,000)	0.7740	(250,000)
density-TC-10 (nouns)	0.5834	(248,000)	0.6066	(249,000)	0.7235	(250,000)	0.7930	(250,000)
density-TC-10 (all)	0.6171	(249,000)	0.6572	(250,000)	0.7391	(250,000)	0.7777	(250,000)
density-TC-20 (nouns)	0.5904	(248,000)	0.6108	(249,000)	0.7200	(250,000)	0.7870	(250,000)
density-TC-20 (all)	0.6144	(249,000)	0.6647	(250,000)	0.7147	(250,000)	0.7690	(250,000)
density-TC-50 (nouns)	0.5874	(248,000)	0.6002	(249,000)	0.6962	(250,000)	0.7613	(250,000)
density-TC-50 (all)	0.6019	(249,000)	0.6520	(250,000)	0.6698	(250,000)	0.7318	(250,000)
density-NN-5 (nouns)	0.5160	(249,000)	0.4931	(249,000)	0.6541	(250,000)	0.6296	(250,000)
density-NN-5 (all)	0.5028	(250,000)	0.5002	(250,000)	0.6311	(250,000)	0.6249	(250,000)
density-NN-10 (nouns)	0.5053	(249,000)	0.4804	(249,000)	0.6608	(250,000)	0.6380	(250,000)
density-NN-10 (all)	0.4944	(250,000)	0.4888	(250,000)	0.6229	(250,000)	0.6185	(250,000)
density-NN-20 (nouns)	0.4779	(249,000)	0.4501	(249,000)	0.6453	(250,000)	0.6397	(250,000)
density-NN-20 (all)	0.4795	(250,000)	0.4684	(250,000)	0.6185	(250,000)	0.6181	(250,000)
density-NN-50 (nouns)	0.4683	(249,000)	0.4247	(249,000)	0.6188	(250,000)	0.6276	(250,000)
density-NN-50 (all)	0.4480	(250,000)	0.4409	(250,000)	0.5815	(250,000)	0.6015	(250,000)
density-TN-5 (nouns)	0.4995	(249,000)	0.4898	(249,000)	0.7325	(250,000)	0.7350	(250,000)
density-TN-5 (all)	0.4921	(250,000)	0.4930	(250,000)	0.7031	(250,000)	0.7224	(250,000)
density-TN-10 (nouns)	0.5005	(249,000)	0.4818	(249,000)	0.7228	(250,000)	0.7246	(250,000)
density-TN-10 (all)	0.4916	(250,000)	0.4885	(250,000)	0.6892	(250,000)	0.7090	(250,000)
density-TN-20 (nouns)	0.4910	(249,000)	0.4655	(249,000)	0.7065	(250,000)	0.7102	(250,000)
density-TN-20 (all)	0.4824	(250,000)	0.4764	(250,000)	0.6685	(250,000)	0.6913	(250,000)
density-TN-50 (nouns)	0.4749	(249,000)	0.4418	(249,000)	0.6665	(250,000)	0.6780	(250,000)
density-TN-50 (all)	0.4641	(250,000)	0.4539	(250,000)	0.6266	(250,000)	0.6595	(250,000)

Table 2. Full results for pair-wise distinctions between degrees of concreteness: nouns.

	window 2				window 20			
	space: N		space: N-V-A		space: N		space: N-V-A	
baseline: frequency	0.5421 (40,000)							
weeds-token OLD	0.5176	(36,966)	0.5543	(38,956)	0.6108	(40,000)	0.6083	(40,000)
weeds-type OLD	0.4771	(36,966)	0.4797	(38,956)	0.5463	(40,000)	0.5723	(40,000)
weeds-token	0.5072	(36,966)	0.5084	(38,956)	0.5163	(40,000)	0.5373	(40,000)
weeds-type	0.5241	(36,966)	0.5270	(38,956)	0.5477	(40,000)	0.5501	(40,000)
entropy	0.5371	(40,000)	0.5280	(40,000)	0.5654	(40,000)	0.5646	(40,000)
density-CC-5 (nouns)	0.4731	(39,800)	0.4212	(39,800)	0.5322	(40,000)	0.5295	(40,000)
density-CC-5 (all)	0.4646	(40,000)	0.4316	(40,000)	0.5058	(40,000)	0.5202	(40,000)
density-CC-10 (nouns)	0.4506	(39,800)	0.3460	(39,800)	0.5115	(40,000)	0.4980	(40,000)
density-CC-10 (all)	0.4148	(40,000)	0.3546	(40,000)	0.4680	(40,000)	0.4883	(40,000)
density-CC-20 (nouns)	0.4059	(39,800)	0.2989	(39,800)	0.4806	(40,000)	0.4556	(40,000)
density-CC-20 (all)	0.3983	(40,000)	0.3212	(40,000)	0.4398	(40,000)	0.4556	(40,000)
density-CC-50 (nouns)	0.3891	(39,800)	0.2427	(39,800)	0.4324	(40,000)	0.3927	(40,000)
density-CC-50 (all)	0.3646	(40,000)	0.2840	(40,000)	0.3698	(40,000)	0.3899	(40,000)
density-TC-5 (nouns)	0.5142	(39,800)	0.5538	(40,000)	0.5697	(40,000)	0.6650	(40,000)
density-TC-5 (all)	0.5139	(40,000)	0.5591	(40,000)	0.6151	(40,000)	0.6475	(40,000)
density-TC-10 (nouns)	0.5142	(39,800)	0.5500	(40,000)	0.5454	(40,000)	0.6381	(40,000)
density-TC-10 (all)	0.5211	(40,000)	0.5613	(40,000)	0.5659	(40,000)	0.6257	(40,000)
density-TC-20 (nouns)	0.5389	(39,800)	0.5664	(40,000)	0.5141	(40,000)	0.6028	(40,000)
density-TC-20 (all)	0.5188	(40,000)	0.5658	(40,000)	0.5289	(40,000)	0.5938	(40,000)
density-TC-50 (nouns)	0.5492	(39,800)	0.5637	(40,000)	0.4870	(40,000)	0.5604	(40,000)
density-TC-50 (all)	0.4932	(40,000)	0.5378	(40,000)	0.4625	(40,000)	0.5292	(40,000)
density-NN-5 (verbs)	0.5925	(40,000)	0.5698	(40,000)	0.5789	(40,000)	0.5454	(40,000)
density-NN-5 (all)	0.5624	(40,000)	0.5756	(40,000)	0.6319	(40,000)	0.6035	(40,000)
density-NN-10 (verbs)	0.6020	(40,000)	0.5436	(40,000)	0.5695	(40,000)	0.5284	(40,000)
density-NN-10 (all)	0.5962	(40,000)	0.6049	(40,000)	0.6319	(40,000)	0.6186	(40,000)
density-NN-20 (verbs)	0.5861	(40,000)	0.5509	(40,000)	0.5353	(40,000)	0.5023	(40,000)
density-NN-20 (all)	0.6048	(40,000)	0.6043	(40,000)	0.6223	(40,000)	0.6075	(40,000)
density-NN-50 (verbs)	0.5832	(40,000)	0.5355	(40,000)	0.4829	(40,000)	0.4409	(40,000)
density-NN-50 (all)	0.6054	(40,000)	0.5813	(40,000)	0.6211	(40,000)	0.5976	(40,000)
density-TN-5 (verbs)	0.5081	(40,000)	0.4818	(40,000)	0.5275	(40,000)	0.5120	(40,000)
density-TN-5 (all)	0.4891	(40,000)	0.4656	(40,000)	0.5586	(40,000)	0.5499	(40,000)
density-TN-10 (verbs)	0.5241	(40,000)	0.4919	(40,000)	0.5206	(40,000)	0.5098	(40,000)
density-TN-10 (all)	0.5128	(40,000)	0.4932	(40,000)	0.5640	(40,000)	0.5605	(40,000)
density-TN-20 (verbs)	0.5260	(40,000)	0.4903	(40,000)	0.5057	(40,000)	0.4972	(40,000)
density-TN-20 (all)	0.5305	(40,000)	0.5167	(40,000)	0.5644	(40,000)	0.5638	(40,000)
density-TN-50 (verbs)	0.5087	(40,000)	0.4762	(40,000)	0.4608	(40,000)	0.4569	(40,000)
density-TN-50 (all)	0.5436	(40,000)	0.5288	(40,000)	0.5548	(40,000)	0.5529	(40,000)

Table 3. Full results for pair-wise distinctions between degrees of concreteness: verbs.

	window 2				window 20			
	space: N		space: N-V-A		space: N		space: N-V-A	
baseline: frequency	0.7276 (86,636)							
weeds-token OLD	0.5110	(38,890)	0.5424	(46,677)	0.5387	(58,382)	0.5382	(60,985)
weeds-type OLD	0.4246	(38,890)	0.4054	(46,677)	0.3845	(58,382)	0.3916	(60,985)
weeds-token	0.7064	(38,890)	0.7141	(46,677)	0.7220	(58,382)	0.7221	(60,985)
weeds-type	0.7167	(38,890)	0.7227	(46,677)	0.7279	(58,382)	0.7250	(60,985)
entropy	0.7068	(49,139)	0.7152	(53,735)	0.7241	(61,152)	0.7244	(62,882)
density-CC-5 (nouns)	0.4138	(42,371)	0.4342	(42,371)	0.4934	(57,062)	0.4895	(57,062)
density-CC-5 (all)	0.3904	(48,114)	0.4016	(48,114)	0.4572	(59,475)	0.4665	(59,475)
density-CC-10 (nouns)	0.4114	(42,371)	0.4293	(42,371)	0.4903	(57,062)	0.4862	(57,062)
density-CC-10 (all)	0.3637	(48,114)	0.3755	(48,114)	0.4487	(59,475)	0.4613	(59,475)
density-CC-20 (nouns)	0.4172	(42,371)	0.4313	(42,371)	0.4823	(57,062)	0.4797	(57,062)
density-CC-20 (all)	0.3612	(48,114)	0.3713	(48,114)	0.4451	(59,475)	0.4556	(59,475)
density-CC-50 (nouns)	0.4381	(42,371)	0.4556	(42,371)	0.4850	(57,062)	0.4844	(57,062)
density-CC-50 (all)	0.3695	(48,114)	0.3806	(48,114)	0.4396	(59,475)	0.4539	(59,475)
density-TC-5 (nouns)	0.4664	(46,866)	0.4569	(47,724)	0.5089	(61,006)	0.5020	(61,016)
density-TC-5 (all)	0.4691	(47,669)	0.4609	(50526)	0.4671	(61,067)	0.4676	(62,775)
density-TC-10 (nouns)	0.4638	(46,866)	0.4498	(47,724)	0.4977	(61,006)	0.4903	(61,016)
density-TC-10 (all)	0.4588	(47,734)	0.4496	(50,526)	0.4449	(61,067)	0.4497	(62,775)
density-TC-20 (nouns)	0.4640	(46,866)	0.4473	(47,724)	0.4954	(61,006)	0.4836	(61,016)
density-TC-20 (all)	0.4534	(47,744)	0.4431	(50,526)	0.4346	(61,067)	0.4408	(62,775)
density-TC-50 (nouns)	0.4649	(46,866)	0.4447	(47,724)	0.4981	(61,006)	0.4846	(61,016)
density-TC-50 (all)	0.4439	(47,744)	0.4317	(50,526)	0.4245	(61,067)	0.4336	(62,775)
density-NN-5 (nouns)	0.4756	(48,770)	0.4934	(53,452)	0.5117	(61,037)	0.5172	(62,797)
density-NN-5 (all)	0.4640	(48,868)	0.4890	(53,517)	0.4857	(61,090)	0.4990	(62,813)
density-NN-10 (nouns)	0.4778	(48,770)	0.4785	(53,456)	0.5187	(61,037)	0.5149	(62,797)
density-NN-10 (all)	0.4753	(48,868)	0.4872	(53,517)	0.4933	(61,090)	0.5017	(62,813)
density-NN-20 (nouns)	0.4679	(48,770)	0.4717	(53,456)	0.5256	(61,037)	0.5141	(62,797)
density-NN-20 (all)	0.4691	(48,868)	0.4801	(53,517)	0.4965	(61,090)	0.4983	(62,813)
density-NN-50 (nouns)	0.4556	(48,770)	0.4576	(53,456)	0.5294	(61,037)	0.5129	(62,797)
density-NN-50 (all)	0.4569	(48,868)	0.4714	(53,517)	0.5021	(61,090)	0.4987	(62,813)
density-TN-5 (nouns)	0.5197	(48,894)	0.5211	(53,564)	0.4676	(61,055)	0.4789	(62,821)
density-TN-5 (all)	0.5283	(48,977)	0.5329	(53,597)	0.4476	(61,108)	0.4663	(62,821)
density-TN-10 (nouns)	0.5019	(48,894)	0.5015	(53,564)	0.4611	(61,055)	0.4708	(62,821)
density-TN-10 (all)	0.5083	(48,977)	0.5156	(53,597)	0.4397	(61,108)	0.4558	(62,821)
density-TN-20 (nouns)	0.4864	(48,894)	0.4810	(53,564)	0.4569	(61,055)	0.4587	(62,821)
density-TN-20 (all)	0.4913	(48,977)	0.4971	(53,597)	0.4340	(61,108)	0.4464	(62,821)
density-TN-50 (nouns)	0.4627	(48,894)	0.4521	(53,564)	0.4494	(61,055)	0.4414	(62,821)
density-TN-50 (all)	0.4677	(48,977)	0.4739	(53,597)	0.4255	(61,108)	0.4318	(62,821)

Table 4. Full results for pair-wise distinctions between degrees of specificity: nouns.

	window 2				window 20			
	space: N		space: N-V-A		space: N		space: N-V-A	
baseline: frequency	0.7110 (39,572)							
weeds-token OLD	0.5158	(27,094)	0.5310	(28,500)	0.5191	(32,686)	0.5273	(33,438)
weeds-type OLD	0.4212	(27,094)	0.4259	(28,500)	0.4038	(32,686)	0.4146	(33,438)
weeds-token	0.7054	(27,094)	0.7083	(28,500)	0.7104	(32,686)	0.7088	(33,438)
weeds-type	0.7111	(27,094)	0.7107	(28,500)	0.7112	(32,686)	0.7095	(33,438)
entropy	0.7039	(30,622)	0.7049	(31,529)	0.7072	(33,704)	0.7068	(34255)
density-CC-5 (nouns)	0.4888	(28,306)	0.4273	(28,372)	0.5149	(32,445)	0.4780	(32,445)
density-CC-5 (all)	0.4972	(29,517)	0.4167	(29,572)	0.5001	(33,241)	0.4750	(33,241)
density-CC-10 (nouns)	0.4813	(28,306)	0.4045	(28,372)	0.5143	(32,445)	0.4751	(32,445)
density-CC-10 (all)	0.4869	(29,517)	0.4005	(29,572)	0.4971	(33,241)	0.4643	(33,241)
density-CC-20 (nouns)	0.4803	(28,306)	0.4067	(28,372)	0.5164	(32,445)	0.4742	(32,445)
density-CC-20 (all)	0.4776	(29,517)	0.4020	(29,572)	0.5027	(33,241)	0.4735	(33,241)
density-CC-50 (nouns)	0.4938	(28,306)	0.4387	(28,372)	0.5213	(32,445)	0.4883	(32,445)
density-CC-50 (all)	0.5017	(29,517)	0.4253	(29,572)	0.5158	(33,241)	0.4907	(33,241)
density-TC-5 (nouns)	0.4500	(30,092)	0.4479	(30,292)	0.4941	(33,704)	0.4869	(33,704)
density-TC-5 (all)	0.4555	(30,292)	0.4582	(30,652)	0.4831	(33,704)	0.4808	(34,251)
density-TC-10 (nouns)	0.4498	(30,092)	0.4467	(30,292)	0.4807	(33,704)	0.4678	(33,704)
density-TC-10 (all)	0.4491	(30,292)	0.4518	(30,652)	0.4615	(33,704)	0.4593	(34,251)
density-TC-20 (nouns)	0.4509	(30,092)	0.4469	(30,292)	0.4789	(33,704)	0.4642	(33,704)
density-TC-20 (all)	0.4428	(30,292)	0.4459	(30,652)	0.4499	(33,704)	0.4440	(34,251)
density-TC-50 (nouns)	0.4506	(30,092)	0.4433	(30,292)	0.4801	(33,704)	0.4631	(33,704)
density-TC-50 (all)	0.4377	(30,292)	0.4359	(30,652)	0.4420	(33,704)	0.4408	(34,251)
density-NN-5 (verbs)	0.5191	(30,602)	0.5230	(31,494)	0.5265	(33,704)	0.5340	(34,251)
density-NN-5 (all)	0.5307	(30,611)	0.5162	(31,508)	0.5562	(33,704)	0.5586	(34,251)
density-NN-10 (verbs)	0.5123	(30,602)	0.5149	(31,494)	0.5166	(33,704)	0.5298	(34,251)
density-NN-10 (all)	0.5288	(30,611)	0.5201	(31,508)	0.5552	(33,704)	0.5625	(34,251)
density-NN-20 (verbs)	0.4941	(30,602)	0.5084	(31,494)	0.5012	(33,704)	0.5173	(34,251)
density-NN-20 (all)	0.5132	(30,611)	0.5169	(31,508)	0.5455	(33,704)	0.5628	(34,251)
density-NN-50 (verbs)	0.4867	(30,602)	0.4933	(31,494)	0.4754	(33,704)	0.4929	(34,251)
density-NN-50 (all)	0.4975	(30,611)	0.5057	(31,508)	0.5315	(33,704)	0.5526	(34,251)
density-TN-5 (verbs)	0.5194	(30,609)	0.5213	(31,508)	0.5047	(33,704)	0.5009	(34,251)
density-TN-5 (all)	0.5731	(30,614)	0.5698	(31,511)	0.5616	(33,704)	0.5420	(34,251)
density-TN-10 (verbs)	0.5056	(30,609)	0.5053	(31,508)	0.4875	(33,704)	0.4895	(34,251)
density-TN-10 (all)	0.5634	(30,614)	0.5596	(31,511)	0.5509	(33,704)	0.5361	(34,251)
density-TN-20 (verbs)	0.4908	(30,609)	0.4909	(31,508)	0.4667	(33,704)	0.4758	(34,251)
density-TN-20 (all)	0.5472	(30,614)	0.5430	(31,511)	0.5363	(33,704)	0.5278	(34,251)
density-TN-50 (verbs)	0.4654	(30,609)	0.4644	(31,508)	0.4356	(33,704)	0.4506	(34,251)
density-TN-50 (all)	0.5222	(30,614)	0.5232	(31,511)	0.5103	(33,704)	0.5149	(34,251)

Table 5. Full results for pair-wise distinctions between degrees of specificity: verbs.
